# The mRNA-binding protein DDX3 mediates TGF-**β**1 upregulation of translation and promotes pulmonary fibrosis

**DOI:** 10.1172/jci.insight.167566

**Published:** 2023-04-10

**Authors:** Wensheng Chen, Darrell Pilling, Richard H. Gomer

**Affiliations:** Department of Biology, Texas A&M University, College Station, Texas, USA.

**Keywords:** Cell Biology, Immunology, Fibrosis, Translation

## Abstract

Pulmonary fibrosis is potentiated by a positive feedback loop involving the extracellular sialidase enzyme neuraminidase 3 (NEU3) causing release of active TGF-β1 and TGF-β1 upregulating NEU3 by increasing translation without affecting mRNA levels. In this report, we elucidate the TGF-β1 upregulation of the translation mechanism. In human lung fibroblasts, TGF-β1 increased levels of proteins, including NEU3, by increasing translation of the encoding mRNAs without significantly affecting levels of these mRNAs. A total of 180 of these mRNAs shared a common 20-nucleotide motif. Deletion of this motif from *NEU3* mRNA eliminated the TGF-β1 upregulation of NEU3 translation, while insertion of this motif in 2 mRNAs insensitive to TGF-β1 caused TGF-β1 to upregulate their translation. RNA-binding proteins including DEAD box helicase 3, X-linked (DDX3), bind the RNA motif, and TGF-β1 regulates their protein levels and/or binding to the motif. We found that DDX3 was upregulated in the fibrotic lesions in patients with pulmonary fibrosis, and inhibiting DDX3 in fibroblasts reduced TGF-β1 upregulation of NEU3 levels. In the mouse bleomycin model of pulmonary fibrosis, injections of the DDX3 inhibitor RK-33 potentiated survival and reduced lung inflammation, fibrosis, and tissue levels of DDX3, TGF-β1, and NEU3. These results suggest that inhibiting an mRNA-binding protein that mediates TGF-β1 upregulation of translation can reduce pulmonary fibrosis.

## Graphical abstract

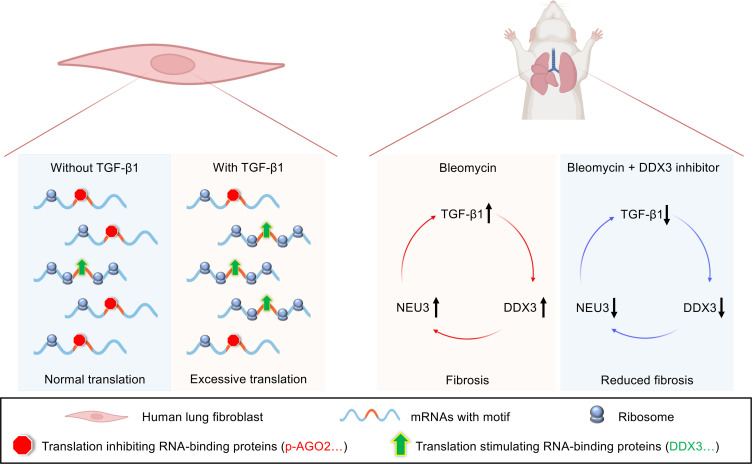

## Introduction

Idiopathic pulmonary fibrosis (IPF) is a chronic and progressive lung disease with an average survival of 3–5 years following diagnosis (1, 2). Fibroblast and extracellular matrix accumulation replacing normal lung tissue in patients with IPF leads to loss of lung function, disruption of gas exchange, and respiratory failure (3–5). The extracellular sialidase neuraminidase 3 (NEU3) is upregulated in fibrotic lesions in IPF patient lungs and the lungs of mice with bleomycin-induced pulmonary fibrosis (6). Mice lacking NEU3 essentially do not develop fibrosis in the bleomycin model, and aspiration of mouse NEU3 (but not enzyme-dead NEU3) causes pulmonary fibrosis in mice (7, 8). Inhibitors of NEU3 significantly reduce fibrosis in the mouse bleomycin model (6, 9). Together, this suggests that NEU3 upregulation potentiates pulmonary fibrosis in mice.

The high extracellular levels of active TGF-β1 drive disease progression by modulating inflammation, fibrosis, cell growth, migration, or changes in cell morphology and/or phenotype (10). High levels of extracellular TGF-β1 are present in the fibrotic lesions in IPF, and the TGF-β1 activates receptors on fibroblasts to cause them to proliferate and increase the production of extracellular matrix, as well as the production of NEU3 (6, 11, 12).

In human cardiac fibroblasts incubated with TGF-β1 and in human dilated cardiomyopathy tissue, RNA-Seq of monosomes, polysomes, and total mRNA indicated that one-third of the proteins regulated by TGF-β1 are regulated at the level of translation (13). We previously found that TGF-β1 increases the amount of *NEU3* mRNA in polysomes and increases NEU3 protein levels, without affecting *NEU3* total mRNA levels in human lung epithelial cells (14). This indicated that TGF-β1 increases levels of NEU3 at the level of translation and not transcription.

To investigate the mechanism of TGF-β1 upregulation of NEU3 translation and test if blocking this effect could attenuate pulmonary fibrosis, in this report, we identified proteins in addition to NEU3 in human lung fibroblasts whose levels are increased by TGF-β1 at the level of translation but not transcription, then identified a common motif in the encoding mRNAs. We then found that the motif is necessary and sufficient for the TGF-β1 upregulation of translation, then identified RNA-binding proteins that bind to, or decrease binding to, the motif in response to TGF-β1. A protein that binds the mRNA motif in response to TGF-β1 is DEAD box helicase 3, X-linked (DDX3) (15), and downregulation of DDX3 or treatment with the DDX3 inhibitors RK-33 and IN-1 reduced TGF-β1–induced NEU3 levels in fibroblasts. In a mouse model of pulmonary fibrosis, RK-33 reduced bleomycin-induced lung inflammation and fibrosis, and lung tissue levels of DDX3, TGF-β1, and NEU3, suggesting that the TGF-β1 upregulation of the translation pathway may be a viable target to inhibit fibrosis.

## Results

### TGF-β1 induces ribosomal mRNA shifts and leads to mRNA/protein mismatches in human lung fibroblasts.

**Figure 1:**
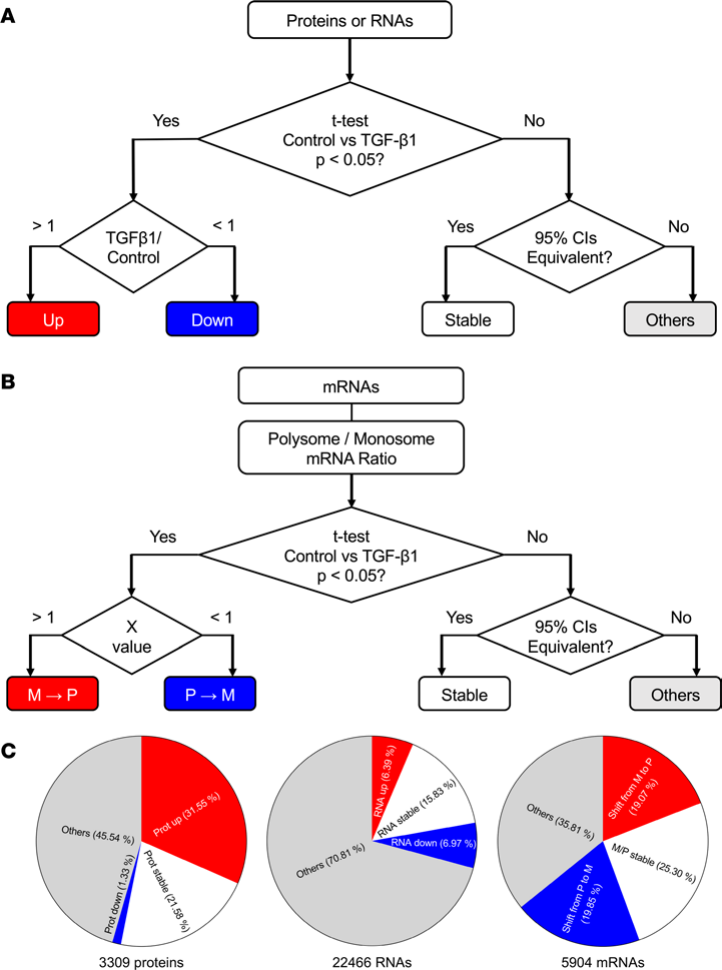
Overview of the data. (**A** and **B**) Strategy of proteomics and RNA-Seq analysis of 4 HLF cell lines treated with or without TGF-β1, starting with *t* tests to compare the effect of TGF-β1 with control for each protein, RNA, or polysome/monosome ratio for an mRNA. In **B**, M→P indicates a TGF-β1–induced shift from monosomes to polysomes (and thus a TGF-β1–induced increased in translation) for an mRNA; P→M indicates a TGF-β1–induced shift from polysomes to monosomes. (**C**) Distribution of protein responses to TGF-β1, distribution of RNA responses to TGF-β1, and distribution of polysome/monosome ratios for mRNAs detected in monosomes and/or polysomes.

To elucidate the effect of TGF-β1 on fibroblasts, 4 human lung fibroblast cell lines were treated with or without recombinant human TGF-β1. Proteomics and RNA-Seq data were analyzed according to the workflow in Figure 1 (Supplemental Data 1–7; supplemental material available online with this article; https://doi.org/10.1172/jci.insight.167566DS1). Proteomics identified 3,309 proteins; TGF-β1 increased levels of 1,044 of these proteins and decreased levels of 44 proteins (*P* < 0.05; multiple 2-tailed *t* tests, *n* = 4); and 714 proteins were statistically equivalent with or without TGF-β1 treatment (Figure 1C). RNA-Seq analysis of these cells identified 22,466 transcripts (a mix of mRNAs and noncoding RNAs), and TGF-β1 increased levels of 1,436 RNAs and decreased levels of 1,567 RNAs (*P* < 0.05; multiple 2-tailed *t* tests, *n* = 4), while 3,556 RNAs were statistically equivalent with or without TGF-β1 treatment (Figure 1C). As we did for lung epithelial cells (14), we fractionated control and TGF-β1–treated fibroblasts to obtain free mRNA, monosome-associated mRNA (henceforth referred to as monosome fraction), and polysome-associated mRNA. As with the analysis of the proteomics and RNA-Seq of total mRNA, multiple *t* tests were used to identify mRNAs with a statistically significant TGF-β1–induced shift from monosomes to polysomes, a shift from polysomes to monosomes, or a statistically significant absence of a shift (Figure 1, B and C). Some protein/mRNA pairs did not show statistical significance for increase, decrease, or no change in one or more of the categories (mRNA, polysome/monosome ratio, or protein) and were thus excluded from further analysis. Combining the data, there were 1,149 proteins with statistical significance (increase, decrease, or no change) for the TGF-β1 effect on mRNA, polysome/monosome ratio, and protein.

The 1,149 proteins were divided into 27 groups according to total protein levels, total mRNA levels, and ribosomal mRNA shifts (Supplemental Table 1). TGF-β1 increased levels of 693 and decreased levels of 27 of the 1,149 proteins. For 545 of the 1,149 proteins, the TGF-β1 effect on the protein level did not match the TGF-β1 effect on the mRNA level (groups 4–12 and 16–24). For the proteins upregulated by TGF-β1, Gene Ontology (GO) analysis indicated that group 1 included extracellular matrix and amino acid biosynthesis. Group 4 (total mRNA stable • translation up • protein up) proteins included 26S protease regulatory subunits, ribosome proteins, and translation initiation and elongation factors. Group 7 (total mRNA down • translation up • protein up) included cellular stress response and autophagy proteins (Supplemental Figure 1). Groups 19–27 (proteins decreased by TGF-β1) all had fewer than 10 proteins and did not show any significant enrichment. Although NEU3 had no proteomics reads, it is a group 4–like protein (TGF-β1 increases NEU3 protein in lung fibroblasts; ref. 6), there was no significant effect of TGF-β1 on total mRNA level (control 26.7 ± 4.5, TGF-β1 24.9 ± 3.6, both in units of relative abundance from the RNA-Seq, *n* = 4); *NEU3* showed a monosome to polysome mRNA shift (X = 5.92, *P* < 0.01). Together, these results suggest that TGF-β1 has a wide and complex effect on both transcription and translation control of human lung fibroblasts.

### Group 4 mRNAs have a common motif in their coding region.

Translation regulation of mRNAs is mediated in general by microRNAs, upstream open reading frames (uORFs), or motifs in the mRNA. Of the 181 proteins in group 4, 152 of the corresponding mRNAs have no known microRNA-binding site, and the remaining 29 mRNAs bind a variety of microRNAs, suggesting that the TGF-β1 regulation of translation that we are looking at may not be microRNA mediated. There were similar percentages of mRNAs with uORFs in 9 representative groups (Supplemental Figure 2, A and B), suggesting that the presence of uORFs may not be the main factor that mediates TGF-β1 regulation of translation. A search for common motifs, using an algorithm that looks for both a common sequence and a common structure of the RNA domain, in each of the 22 groups in Supplemental Table 1 with more than 3 proteins in the group, identified common motifs in 14 of the groups. Interestingly, the common motifs of the groups where TGF-β1 affected translation (groups 1, 3, 4, 6, and 7) were mainly located in the coding sequence (CDS) of the mRNAs, while the common motifs in the groups where TGF-β1 had no significant effect on translation (groups 2, 5, 14, and 17) tended to be in the UTRs (Supplemental Figure 2C). In group 4, mRNAs (179 of 181) contained a common motif at *P* < 10^–6^ (Supplemental Table 1), and 137 of the 179 had the motif in the CDS. *NEU3* mRNA had 2 group 4 motifs in its CDS: 5′ CCTGAAGCCACTGATGGAAG 3′ (315 to 334 in the CDS) and 5′ ACTGAGGCTGGAGGAGGAAG 3′ (1,041 to 1,060 in the CDS). The group 4 common motif was observed in a small percentage of mRNAs in other groups (Supplemental Figure 2D), and the 2 other groups with the highest percentage of mRNAs containing a group 4 motif (groups 1 and 13) also had a TGF-β1–induced mRNA monosome to polysome shift. These results indicate that the common motif may be associated with the TGF-β1–induced translation.

### The motifs in NEU3 mRNA are necessary for TGF-β1–increased NEU3 translation.

**Figure 2:**
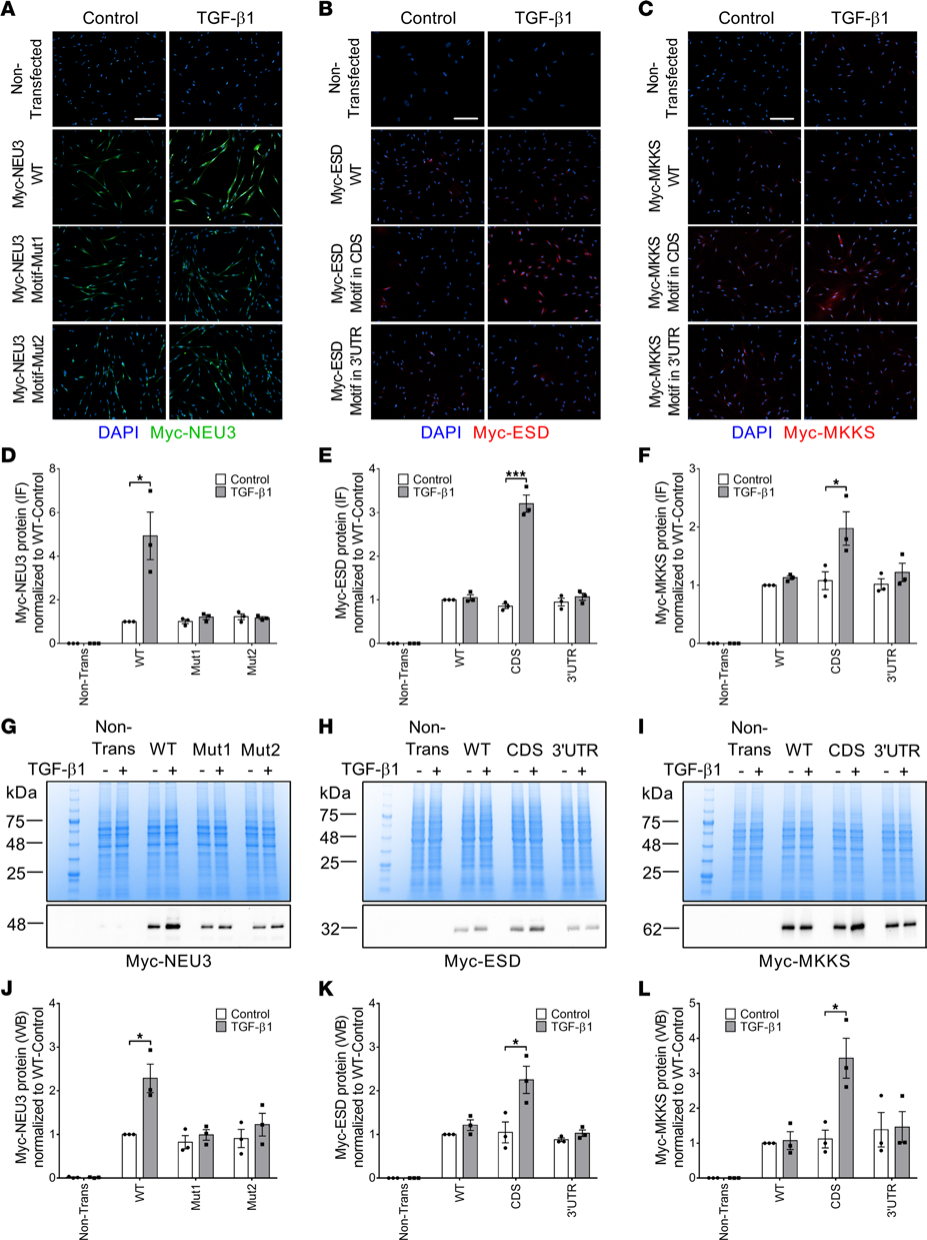
The motifs in the *NEU3* mRNA are necessary for TGF-β1–increased NEU3 translation, and the group 4 motif is sufficient for TGF-β1–induced translation of *S*-formylglutathione hydrolase and McKusick-Kaufman syndrome protein. (**A**) Human lung fibroblasts transfected with the indicated constructs were cultured without (control) or with TGF-β1 and then stained with anti-Myc-tag antibodies. Green indicates Myc-NEU3 staining and blue is DAPI counterstain. Bar is 100 μm. Images are representative of 3 independent experiments. (**B** and **C**) Human lung fibroblasts transfected with the indicated constructs were cultured without (control) or with TGF-β1 and then stained with anti–Myc-tag antibodies. Red indicates Myc-ESD or Myc-MKKS staining and blue is DAPI counterstain. Images are representative of 3 independent experiments. (**D**–**F**) Quantification of **A**–**C**. IF, immunofluorescence. (**G**) Myc-NEU3 in transfected and nontransfected human lung fibroblasts was confirmed by Western blotting with an anti–Myc-tag antibody. Top image is Coomassie blue–stained gel of cell lysates; bottom image is Western blot. (**H** and **I**) Myc-ESD and Myc-MKKS in transfected and nontransfected human lung fibroblasts were verified by Western blotting with an anti–Myc-tag antibody. Images in **G**–**I** are representative of 3 independent experiments. (**J**–**L**) Quantification of **G**–**I**. WB, Western blot. Values in **D**–**F** and **J**–**L** are mean ± SEM, *n* = 3. * *P* < 0.05, *** *P* < 0.001 (*t* tests). ESD, *S*-formylglutathione hydrolase; MKKS, McKusick-Kaufman syndrome protein.

To test the hypothesis that the group 4 motifs in *NEU3* mRNA are necessary for TGF-β1 upregulation of NEU3 translation, 3 Myc-tagged NEU3 expression plasmids were made. Myc-NEU3-WT contains the NEU3 mRNA CDS with a Myc tag inserted at the 3′ end of the protein coding region, with the 2 group 4 motifs in the *NEU3* CDS. The construct did not contain the *NEU3* 5′ or 3′UTRs. In the Myc-NEU3-Motif-Mut1 plasmid, the first motif was mutated without causing a frameshift, and NEU3-Motif-Mut2 had an intact motif 1 but a disruption of the second motif, again without causing a frameshift (Supplemental Data 8). The plasmids were transfected into human lung fibroblasts, and the cells were treated with or without TGF-β1. Staining with anti-Myc antibodies indicated that the Myc tag was not detectable in nontransfected cells (Figure 2, A, D, G, and J). TGF-β1 increased levels of Myc-NEU3 protein encoded with an mRNA containing the motifs but not Myc-NEU3 encoded with an mRNA with a mutation in either motif (Figure 2, A, D, G, and J). TGF-β1 did not significantly affect total levels of the *Myc-NEU3* mRNAs (Supplemental Figure 3A), but did cause WT *Myc-NEU3* mRNA to shift from monosomes to polysomes, and this effect was abolished when either NEU3 motif was mutated (Supplemental Figure 3D). Another plasmid, Myc-NEU3-Motif-Mut3, with 3 silent mutations (the mutations do not cause any amino acid change) in the first motif (Supplemental Data 8) was constructed and transfected into cells. Again, TGF-β1 increased levels of Myc-NEU3 protein encoded with an mRNA containing the motifs but not Myc-NEU3 encoded with an mRNA with silent mutations in the motif (Supplemental Figure 3, G and H). Taken together, both of the group 4 motifs in *NEU3* mRNA are necessary for the TGF-β1–induced *NEU3* translation.

### The group 4 motif is sufficient for TGF-β1–induced translation.

To determine if the group 4 motif is sufficient for TGF-β1–induced translation, we examined ESD, a protein from group 14 where TGF-β1 did not significantly affect protein levels, mRNA levels, or mRNA translation. As expected, the group 4 motif was not found in the *ESD* mRNA with MEME (16). As above, we transformed human lung fibroblasts with constructs encoding Myc-tagged ESD, Myc-tagged ESD encoded by mRNA with the representative group 4 motif (GGAGGAGGAGGAGGAGGAGG) inserted in the CDS without causing a frameshift, and Myc-tagged ESD encoded by mRNA with the group 4 motif inserted in the 3′UTR. TGF-β1 had no significant effect on ESD protein levels when the motif was absent but increased Myc-ESD protein levels when the motif was in the *Myc-ESD* CDS but not the *Myc-ESD* 3′UTR (Figure 2, B, E, H, and K). TGF-β1 did not significantly affect total levels of *Myc-ESD* mRNA (Supplemental Figure 3B) and did not cause *Myc-ESD* mRNA (with the WT *ESD* CDS) to shift from monosomes to polysomes (Supplemental Figure 3E). When the motif was inserted in the ESD CDS but not the 3′UTR, TGF-β1 caused *Myc-ESD* mRNA to shift from monosomes to polysomes (Supplemental Figure 3E). Similar results, including an effect of the motif in the CDS but not in the 3′UTR, were obtained using MKKS, a protein in the same group as ESD (Figure 2, C, F, I, and L, and Supplemental Figure 3, C and F). Together, these results indicate that inserting the group 4 motif in the CDS of an mRNA is sufficient for TGF-β1–induced translation and that the motif does not potentiate translation when it is in the 3′UTR of an mRNA.

### Changes in polysome/monosome ratios for stable mRNAs can predict changes in proteins in human lung fibroblasts and mouse lungs.

To further check the hypothesis that monosome/polysome shifts in mRNAs affect protein levels, we checked 8 protein/mRNA pairs. Four of these showed TGF-β1–induced monosome to polysome mRNA shifts (Supplemental Table 2). Of these 4, 2 showed a TGF-β1–induced decrease in mRNA levels but an increase in protein levels. For the other 2, the mRNA levels were stable, and the protein levels were too low to show up in the proteomics data (Supplemental Table 2). An additional 4 showed polysome to monosome mRNA shifts and no change in mRNA levels. Two of these showed a TGF-β1–induced decrease in protein levels, while the other 2 had no proteomics reads (Supplemental Table 2). Protein expression of the 8 mRNAs was checked in TGF-β1–treated human lung fibroblasts (HLFs) by staining Western blots and by staining bleomycin-treated mouse lung tissues at day 10 (TGF-β1 levels are high in the lungs in the bleomycin mouse model at this time point (Supplemental Figure 4, C and D). TGF-β1 increased levels of 3 of the 4 monosome to polysome mRNA shift proteins in HLFs (the anti–microtubule cross-linking factor 1 [anti-MTCL1] antibody did not show staining of Western blots), and all 4 showed upregulation in bleomycin-treated mouse lungs (Supplemental Figure 4A). Similarly, TGF-β1 decreased levels of 3 of the 4 polysome to monosome mRNA shift proteins in HLFs (the anti–anaphase-promoting complex subunit 4 [anti-APC4] antibody did not show staining of Western blots), and all 4 showed downregulation in bleomycin-treated mouse lungs (Supplemental Figure 4E). The TGF-β1–induced increases of polysome/monosome (PM) ratios of group 4 mRNAs were positively correlated with the TGF-β1–induced changes in the corresponding protein levels (Supplemental Figure 5). These results suggest that data from RNA-Seq of monosomes and polysomes, in addition to RNA-Seq of total RNA, can increase the ability to predict (albeit without 100% certainty) the effects of TGF-β1 on protein levels.

### TGF-β1 regulates group 4 motif–interacting RBPs, and one of the RBPs, DDX3, increases in human IPF lungs.

**Figure 3:**
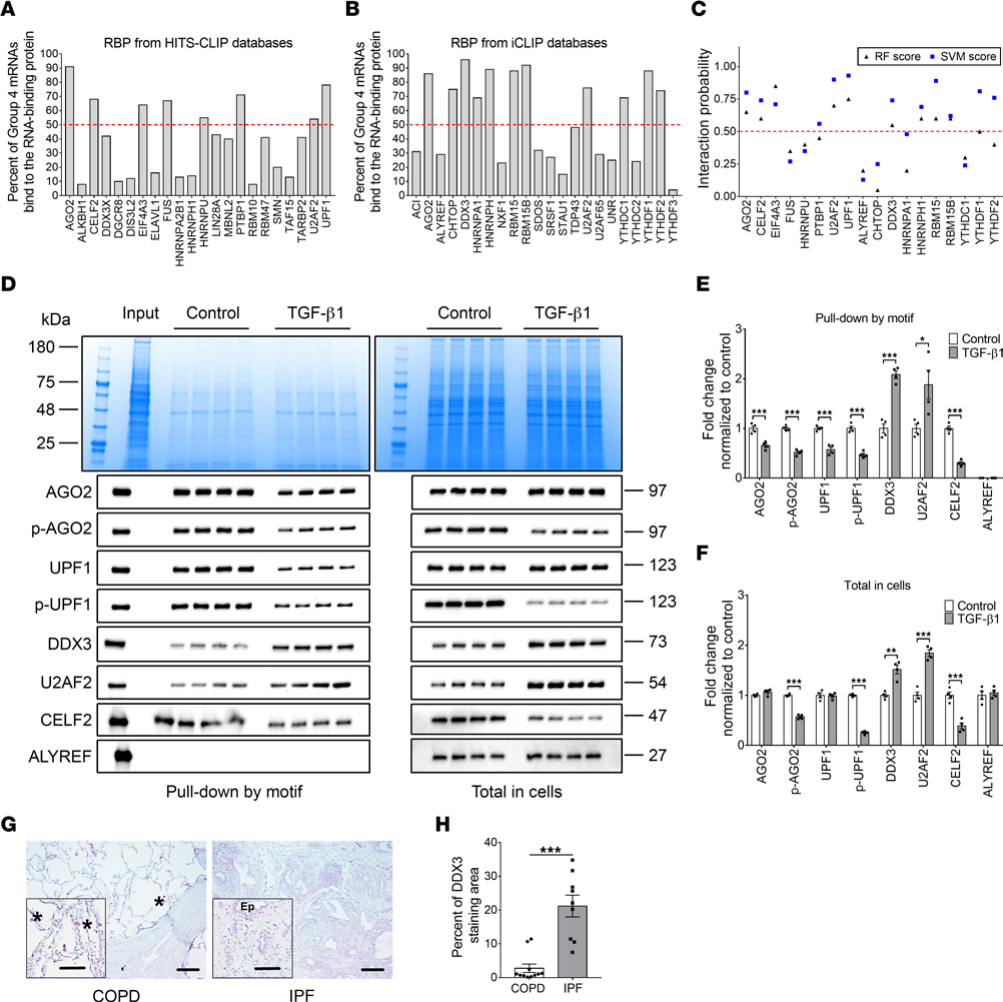
TGF-β1 regulates group 4 motif–interacting RBPs, and one of the RBPs, DDX3, is upregulated in human IPF lungs. (**A** and **B**) RBPs that can bind to group 4 mRNAs were identified from the HITS-CLIP database and the iCLIP database. Red dashed line indicates 50%. (**C**) Group 4 motif–specific RBPs were selected by RNA-protein interaction prediction software (RPISeq). The interaction probability was predicted and scored by a Random Forest classifier (triangles) and a Support Vector Machine classifier (squares). RBPs with scores higher than 0.5 (red dashed line) were considered as having a high possibility of interacting with the indicated RBP. (**D**) Left: The biotin-tagged RNA motif was used to pull down material from control and TGF-β1–treated cells. The Coomassie-stained gel shows the material in the pull-down samples, and the Western blots show staining of the samples with the indicated antibodies. Right: The Coomassie-stained gel shows total cell lysate, and the Western blots show staining of the total cell lysate with the indicated antibodies. (**E** and **F**) Quantification of protein levels in pull-down samples and total samples. Values in **E** and **F** are mean ± SEM, *n* = 4. * *P* < 0.05, ** *P* < 0.01, *** *P* < 0.001 (*t* tests). (**G**) Lung tissue sections from patients with COPD or IPF were stained with anti-DDX3 antibodies. Left: Section from a COPD patient with FVC > 80%. Right: Section from a patient with IPF with FVC < 50%. Positive staining was identified by red color, and nuclei are counterstained blue. Bar is 200 μm. Inserts indicate higher magnification images. Bar is 100 μm. Asterisks indicate alveolar macrophages and Ep indicates epithelial vessels. Images are representative of 11 COPD and 9 ILD patients per group. (**H**) The percentage area of lung tissue stained by DDX3 antibody. Values are mean ± SEM, *n* = 9–11. *** *P* < 0.01 (*t* test).

UV cross-linking and immunoprecipitation (CLIP) identifies the RNAs in a cell line or tissue that bind to a specific RNA-binding protein (RBP) (17). To elucidate how TGF-β1 induces the motif-dependent translation of group 4 mRNAs, we analyzed published CLIP-Seq databases to identify RBPs that bind group 4 mRNAs (18, 19). Many RBPs that bound to group 4 mRNAs were found, and 17 of them bound to more than 50% of the 182 group 4 mRNAs (Figure 3, A and B). To select the group 4 motif–specific RBPs, an interaction prediction between the group 4 motif and the 17 RBPs was done using RNA-Protein Interaction Prediction (RPISeq) (20) (Figure 3C). For further analysis, we chose argonaute 2 (AGO2), regulator of nonsense transcripts 1 (UPF1), DDX3, U2AF 65 kDa subunit (U2AF2), and CUGBP Elav-like family member 2 (CELF2), as these are known translation regulators, and showed high interaction probabilities with the group 4 motif. AGO2 and UPF1 are P-body proteins, and phosphorylation of AGO2 and UPF1 leads to mRNA translational repression (21, 22). DDX3 is a translation stimulator (23, 24). U2AF2 plays an important role in RNA splicing (25). CELF2 is a translation suppressor (26). Aly/REF export factor (ALYREF), an RBP that binds relatively few group 4 mRNAs (none by HITS-CLIP and few from the iCLIP database; Figure 3, A and B), and which has a low predicted binding to the group 4 motif (Figure 3C), was chosen as a negative control.

To determine if the interaction of these proteins with the motif is regulated by TGF-β1, 5′ biotin–labeled GGAGGAGGAGGAGGAGGAGG RNA (the group 4 consensus motif) was used for pull-down assays with streptavidin-conjugated beads from lysates of 4 different HLF lines cultured with or without TGF-β1. Using antibodies against all of the above RBPs, and antibodies against phosphorylated AGO2 and UPF1 (phospho-AGO2 and phospho-UPF1), we observed that TGF-β1 decreased the interaction of the tagged motif with AGO2, phospho-AGO2, UPF1, phospho-UPF1, and CELF2 and increased the motif binding to DDX3 and U2AF2 (Figure 3, D and E). The software predicted that the RBP ALYREF would not bind the motif, and we indeed observed that the tagged motif did not pull down this protein (Figure 3, D and E). Using the antibodies to stain Western blots of whole cell lysates, we observed that TGF-β1 did not significantly change levels of AGO2, UPF1, or ALYREF; decreased levels of phospho-AGO2, phospho-UPF1, and CELF2; and increased levels of DDX3 and U2AF2 (Figure 3, D and F). The *DDX3* mRNA has a group 4 motif, and is in group 4, suggesting that DDX3 may potentiate its own upregulation in response to TGF-β1. These results suggest that TGF-β1 regulates at least 5 RBPs, some of which may in turn regulate *NEU3* translation.

To determine if DDX3 is associated with human lung fibrosis, we examined the distribution of DDX3 in lung tissue from chronic obstructive pulmonary disease (COPD) patients with relatively normal lungs (>80% forced vital capacity; FVC) and IPF patients with advanced disease (<50% FVC) (Supplemental Table 3). Lung tissue from patients with COPD had limited DDX3 staining except for occasional single positive cells in the alveolar space (Figure 3G). In the lung tissue from patients with IPF, DDX3 was distributed throughout the tissue, including the lung epithelium, leukocytes, and fibrotic areas (Figure 3G). The IPF lungs had a greater area of tissue DDX3 staining than the COPD lungs (Figure 3H). Together, these data indicate that the levels of DDX3 are increased in human lung fibrosis.

Because DDX3 is in group 4, to determine if the increased DDX3 in the pull-down samples was caused by an increased ability of DDX3 to bind the motif, HLFs were treated with TGF-β1 for a short time (0.5, 1, and 2 hours), and lysates of these cells were used for pull-down assays with the motif. DDX3 from the 1- and 2-hour TGF-β1–treated cells showed an increased interaction with the motif, while the total DDX3 levels were not changed (Supplemental Figure 6). These data suggest that TGF-β1 increases the binding of DDX3 to the group 4 motif.

### DDX3 siRNA and DDX3 inhibitors block TGF-β1–induced NEU3 expression in HLFs.

**Figure 4:**
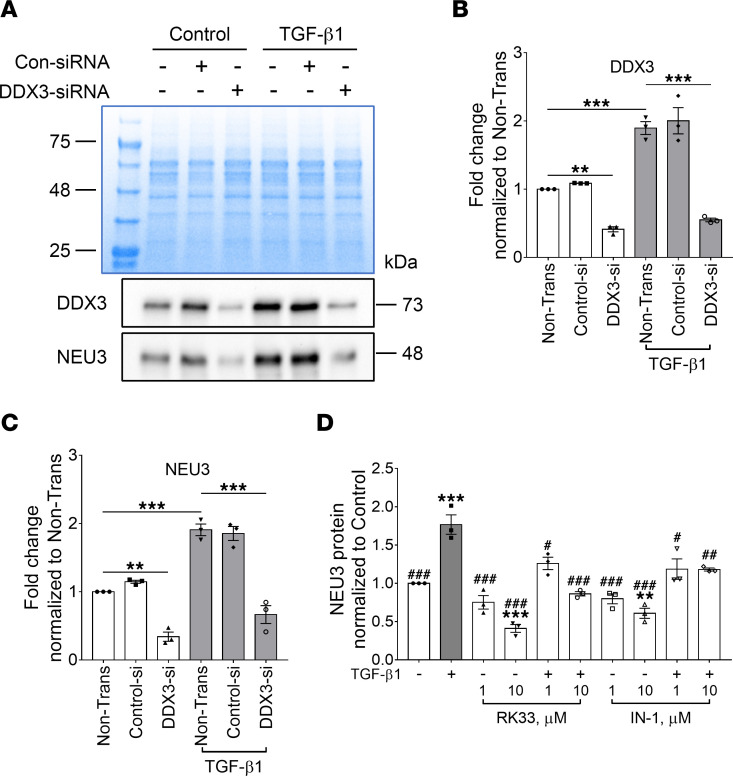
DDX3 siRNA and DDX3 inhibitors block TGF-β1–increased NEU3 levels. (**A**) HLFs were transfected with control or DDX3 siRNA, cultured without (control) or with TGF-β1, and then stained with anti-DDX3 and NEU3 antibodies. Protein levels were determined by Western blotting. Top image is a Coomassie-stained gel of cell lysates; bottom images are Western blots. Images are representative of 3 independent experiments. (**B** and **C**) Quantification of **A**. (**D**) Human lung fibroblasts were treated with DDX3 inhibitors, cultured without (control) or with TGF-β1, and then stained with anti-NEU3 antibody. Protein levels were determined by Western blotting. In **B**–**D**, values are mean ± SEM, *n* = 3. ** *P* < 0.01, *** *P* < 0.001 versus control, ^#^ *P* < 0.05, ^##^ *P* < 0.01, ^###^ *P* < 0.001 versus TGF-β1 treated (1-way ANOVA, Dunnett’s test).

To determine if DDX3 mediates the effect of TGF-β1 on NEU3 translation, HLFs were transfected with DDX3 siRNA or negative control siRNA. HLFs were treated with or without 10 ng/mL TGF-β1 24 hours later. After 48 hours, the culture medium was removed, and cells were lysed, collected, and assessed by Western blotting. Compared with nontransfected cells, DDX3 siRNA decreased DDX3 and NEU3 (Figure 4, A–C). As shown above, TGF-β1 increased DDX3 and NEU3 levels, and DDX3 siRNA blocked the TGF-β1–induced DDX3 and NEU3 upregulation. Negative control siRNA did not decrease DDX3 or NEU3 (Figure 4, A–C). We also determined if 2 DDX3 inhibitors (RK-33 and IN-1) (15, 27) could reduce TGF-β1–mediated NEU3 levels. RK-33 at 10 μM inhibits DDX3 in medulloblastoma and breast cancer cell lines (27, 28). IN-1 at 50 μM inhibits DDX3 in PBMCs and hepatocellular carcinoma cells (15). Compared with control, TGF-β1 increased NEU3 levels (Figure 4D). At 10 μM, both inhibitors reduced NEU3 in cells cultured in the absence of TGF-β1 (Figure 4D), and at 1 and 10 μM, both inhibited TGF-β1–induced upregulation of NEU3 (Figure 4D). These data indicate that DDX3 mediates the effect of TGF-β1 on NEU3.

### RK-33 attenuates pulmonary fibrosis in mice.

**Figure 5:**
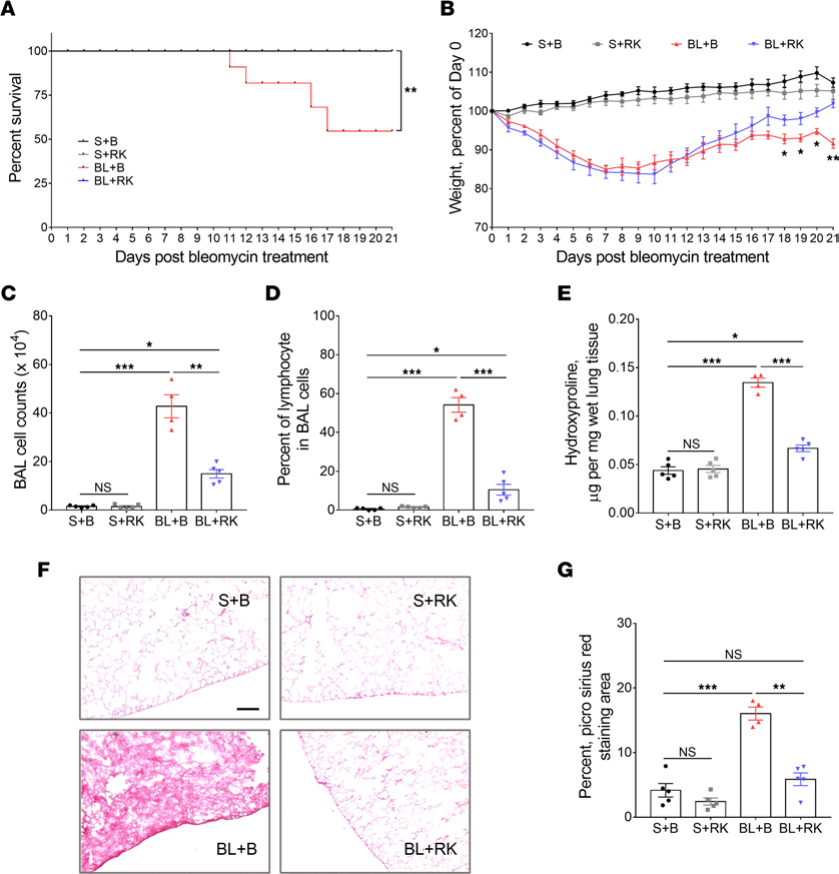
RK-33 attenuates pulmonary fibrosis in mice. Mice were treated with saline or bleomycin, and then starting at day 10 after saline or bleomycin, mice were given 20 mg/kg RK-33 or buffer control every 2 days. Groups are saline with buffer (S+B), saline with RK-33 (S+RK), bleomycin with buffer (BL+B), and bleomycin with RK-33 (BL+RK). (**A**) Survival of mice. *n* = 5 mice for S+B, S+RK, and BL+RK. *n* = 8 mice for BL+B. ** *P* < 0.01 vs. BL+B (Mantel-Cox test). (**B**) Percentage change in body weight. Values are means ± SEM, *n* = 5 mice for S+B, S+RK, and BL+RK, *n* = 4 mice that survived to day 21 for BL+B. * *P* < 0.05, ** *P* < 0.01, BL+B vs. BL+RK (*t* test). (**C**) Total number of cells detected in mouse bronchoalveolar lavage fluid (BALF). (**D**) Percentage of lymphocytes in the BAL cell population as assessed by Wright-Giemsa staining. (**E**) Hydroxyproline levels in mouse lung. (**F**) Picrosirius red–stained mouse lung sections. Bar is 100 μm. Images are representative of 5 mice for S+B, S+RK, and BL+RK, and 4 surviving mice for BL+B. (**G**) Quantification of Picrosirius red–positive stain area in 3 randomly chosen areas of each mouse lung. For **C**–**E** and **G**, values are mean ± SEM, *n* = 5 mice for S+B, S+RK, and BL+RK, *n* = 4 mice for BL+B. * *P* < 0.05, ** *P* < 0.01, *** *P* < 0.001 (1-way ANOVA, Dunnett’s test).

Genetic deletion of NEU3 or inhibiting NEU3 inhibits pulmonary fibrosis in mice (6, 7, 9), and as described above, DDX3 inhibitors inhibit TGF-β1–induced upregulation of NEU3 in lung fibroblasts. RK-33 has been used in 2 mouse models of lung cancer and a mouse model of medulloblastoma, at doses between 20 and 50 mg/kg, and in conjunction with radiation reduces tumor size in these models. Mice receiving 20 mg/kg RK-33 for 7 weeks show no discernable morphological changes in brain, kidney, liver, or spleen tissue and no significant changes in hematological or serum chemistry parameters, including hemoglobin, blood counts, alanine aminotransferase, aspartate aminotransferase, alkaline phosphatase, blood urea nitrogen, creatinine, albumin, and lipids (29). To determine if RK-33 might inhibit bleomycin-induced pulmonary fibrosis in mice, C57BL/6 mice were treated with an oropharyngeal aspiration of saline or bleomycin, and then starting 10 days after saline or bleomycin, were given intraperitoneal injections of 20 mg/kg RK-33 or buffer control every 2 days. Compared with bleomycin-treated mice, mice that received bleomycin and were then treated with RK-33 had a higher survival rate (Figure 5A). In saline-treated mice, RK-33 did not affect survival or significantly affect body weights (Figure 5, A and B) or heart, kidney, liver, spleen, brown fat, or white fat weights as a percentage of total body weight (Supplemental Figure 7). As previously observed, compared with saline-treated mice, bleomycin-treated mice lost weight over the first 10 days and did not recover their initial weight by day 21 (Figure 5B). RK-33 attenuated the bleomycin-induced weight loss from days 18 to 21 (Figure 5B).

Bleomycin upregulates inflammatory cell counts in mouse lungs. Saline-treated control mice and saline-treated mice that received RK-33 showed similar bronchoalveolar lavage (BAL) cell counts and percentage of lymphocytes in the BAL (Figure 5, C and D). Compared with saline-treated mice, bleomycin-treated control mice showed increased BAL cell counts with an increased percentage of lymphocytes (Figure 5, C and D). Compared with the bleomycin-control mice, bleomycin-treated and RK-33–injected mice had fewer BAL cells; there was a lower percentage of lymphocytes in the BAL.

To determine whether RK-33 injections can decrease bleomycin-induced fibrosis, lung sections were stained with Picrosirius red to detect collagen, and hydroxyproline levels were measured in the lung tissue lysates. Saline-treated control mice and saline-treated mice that received RK-33 showed similar hydroxyproline levels and Picrosirius red staining (Figure 5, E–G). Compared with saline-treated mice, bleomycin-treated mice had increased levels of hydroxyproline and increased lung tissue staining for Picrosirius red (Figure 5, E–G). Compared with the bleomycin control mice, bleomycin-treated and RK-33–injected mice had reduced levels of hydroxyproline and reduced lung tissue staining for Picrosirius. Together, these results indicate that RK-33 can attenuate lung fibrosis in mice.

**Figure 6:**
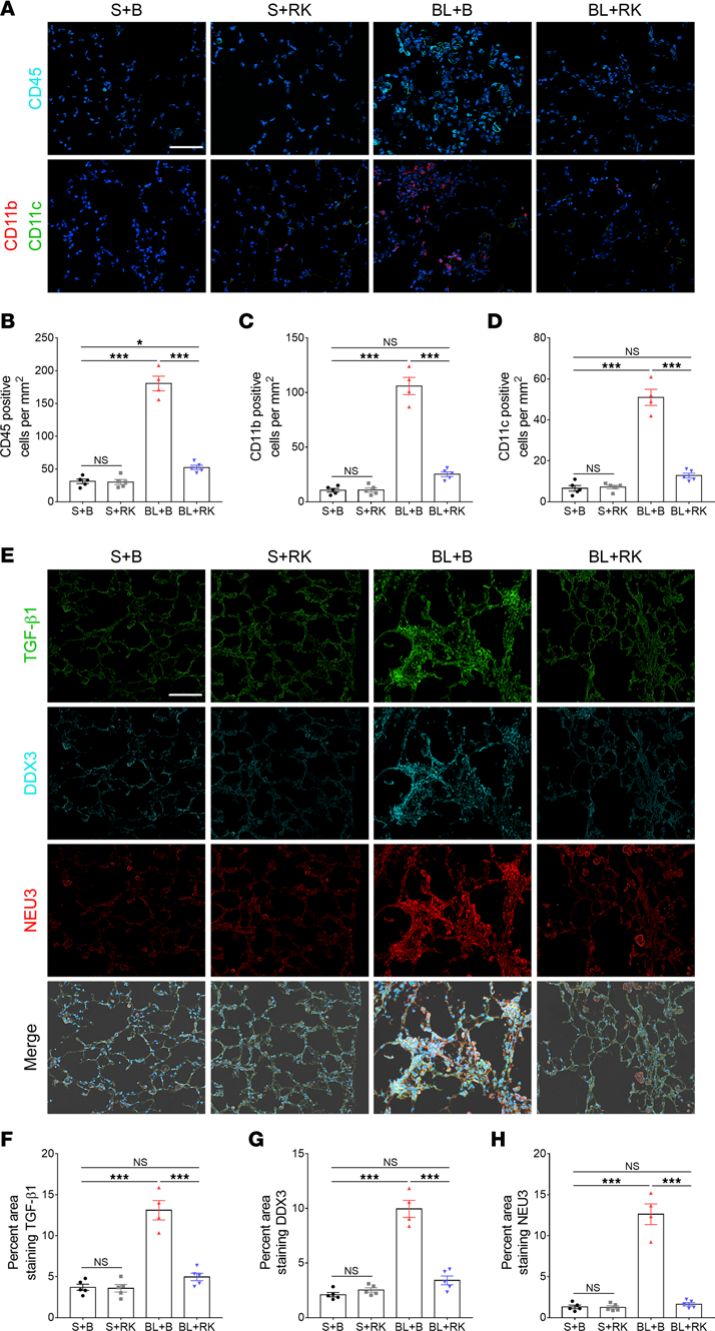
RK-33 reduces bleomycin-increased CD45, CD11b, CD11c, TGF-β1, DDX3, and NEU3. (**A**) Cryosections of mouse lungs from the experiment were stained with anti-CD45, -CD11b, or -CD11c antibodies. Cyan indicates CD45-, red indicates CD11b-, and green indicates CD11c-positive stain. Nuclei are stained blue with DAPI. Bar is 100 μm. Images are representative of *n* = 5 mice for S+B, S+RK, and BL+RK and *n* = 4 mice for BL+B. (**B**–**D**) Quantification of positively stained cells. (**E**) Cryosections of mouse lungs from the experiment were stained with anti–TGF-β1, -DDX3, and -NEU3 antibodies. Cyan indicates DDX3^+^, red indicates NEU3^+^, and green indicates TGF-β1^+^ stain. Nuclei are stained blue with DAPI. Bar is 100 μm. Images are representative of *n* = 5 mice for S+B, S+RK, and BL+RK, *n* = 4 mice for BL+B. (**F**–**H**) Quantification of positively stained area in 3 randomly chosen fields of view for each section from each mouse. Values are mean ± SEM. *** *P* < 0.001 (1-way ANOVA, Dunnett’s test).

Bleomycin aspiration also causes an increase in the number of inflammatory cells and an increase in the levels of TGF-β1 and NEU3 in lung tissue (6, 7, 30). Compared with saline-treated mice, bleomycin increased the number of CD45^+^, CD11b^+^, and CD11c^+^ cells remaining in the lungs after BAL (Figure 6, A–D). RK-33 injections did not affect the counts of CD45^+^, CD11b^+^, and CD11c^+^ cells in mice treated with saline at day 0, but RK-33 significantly reduced the counts of these cells after bleomycin (Figure 6, A–D).

Compared with saline-treated mice, bleomycin increased the expression of TGF-β1, DDX3, and NEU3 in the lung tissue after BAL (Figure 6, E–H). RK-33 injections significantly reduced the levels of all 3 proteins in lung tissue after bleomycin treatment (Figure 6, E–H). The saline-treated control mice and saline-treated mice that received RK-33 showed similar levels of TGF-β1, DDX3, and NEU3 staining (Figure 6, E–H). Together, these data indicate that RK-33 may reduce bleomycin-induced inflammation, fibrosis, and TGF-β1, DDX3, and NEU3 upregulation in mouse lungs.

## Discussion

In both bacteria and eukaryotes, typically only approximately 40% of changes in levels of proteins can be explained by a corresponding change in levels of the associated mRNA (31, 32), indicating that regulating mRNA translation and/or protein degradation plays a more significant role in regulating protein levels than does regulating mRNA levels. In agreement with these observations, we observed that a large fraction of TGF-β1–induced changes in levels within a set of 1,149 proteins in human lung fibroblasts cannot be explained by a corresponding change in the levels of the mRNA encoding the protein. In addition to NEU3, there are at least 181 other proteins in the group 4 subset where TGF-β1 shows a statistically significant absence of a change in mRNA levels but increases translation of the mRNA and increases levels of the protein. Although in lung epithelial cells TGF-β1 decreases NEU3 degradation in addition to increasing *NEU3* translation (14), at least some of the TGF-β1–induced increases in group 4 proteins may be due to increased translation of their mRNAs. In addition, the data indicate that TGF-β1 increases levels of NEU3 in at least 2 different lung cell types by increasing *NEU3* translation without changing levels of *NEU3* mRNA.

In addition to *NEU3*, all but 2 of the group 4 mRNAs contained a common 20-nucleotide motif. Altering the motif in NEU3, and adding the motif to 2 different group 14 mRNAs (where TGF-β1 did not appear to affect mRNA, translation, and protein levels) indicated that the motif is necessary and sufficient for TGF-β1 upregulation of NEU3 translation. The absence of the motif from 2 of the group 4 mRNAs suggests that either the software analysis was too stringent and did not detect a group 4–like motif in these 2 mRNAs or there is some additional mechanism that mediates TGF-β1–induced increased translation of these mRNAs.

Gene expression in eukaryotes is extensively controlled at the posttranscriptional level by RBPs and ribonucleoprotein complexes modulating the stability, transport, editing, and translation of mRNAs (32, 33). We observed that at least 5 RBPs bind the group 4 consensus motif, and all 5 showed a regulation by TGF-β1. AGO2 and UPF1 are known P-body proteins, and the phosphorylation of AGO2 and UPF1 leads to mRNA translational repression (21, 22, 34). TGF-β1 had no significant effect on levels of these 2 RBPs in cells, but rather induced their dephosphorylation and caused them to decrease their interaction with the group 4 motif. CELF2 is a translation suppressor, and TGF-β1 decreased levels of this protein in cells and possibly as a result decreased the amount of binding of this protein to the group 4 motif. Together, these results suggest a scenario where 3 RBPs bind to, and inhibit translation of, group 4 mRNAs. In this scenario, TGF-β1 causes these 3 translation-inhibiting RBPs to decrease their binding to group 4 mRNAs (for 2 of the proteins, apparently by inducing a dephosphorylation of the RBPs), allowing the group 4 mRNAs to increase their interaction with, or ability to be translated by, ribosomes.

The ATP-dependent RNA helicase DDX3 is a translation stimulator (23, 24), and the splicing factor U2AF2 plays a role in RNA splicing (25). TGF-β1 increased levels of both of these 2 RBPs in cells and possibly as a result increased the amount of these 2 RBPs’ binding to a tagged group 4 consensus motif. In the above scenario, TGF-β1–induced increases in levels of the 2 RBPs, 1 of which is a known translation stimulator, would increase their ability to compete with the 3 translation-inhibiting RBPs for binding to the group 4 motif, and the net result would be a TGF-β1–induced increase in translation of group 4 mRNAs. As with the regulation of cyclin-dependent kinases, having multiple positive and negative regulators would allow multiple signals and events in a cell to regulate group 4 mRNA translation with specific dominance relationships.

*NEU3* mRNA contains 2 of the group 4 motifs, and altering either of these abolished the ability of TGF-β1 to increase *NEU3* translation. This suggests that for NEU3, there is an unknown interaction of the motifs, and/or the proteins that bind the motifs, such that the presence of both motifs is necessary for the ability of TGF-β1 to increase *NEU3* translation.

Due to the limitations of the proteomics, we were able to examine the regulation of only the most prevalent proteins in human lung fibroblasts. As others have found in other systems, RNA-Seq of total RNA (31, 32) is a poor predictor of whether a specific signal, in this case TGF-β1, causes an increase or decrease of a protein encoded by an mRNA. Fractionation of mRNA into monosomes and polysomes is also a poor predictor, as exemplified by the 144 proteins in group 13, where there was a clear increase in translation but no increase in protein levels. For unknown reasons, the combination of a group 4 motif and a monosome to polysome shift was a good predictor of an increase in protein levels, at least within the test group, and an intriguing possibility is that combining motif analysis with RNA-Seq of monosomes and polysomes may increase the ability to predict the effects of a signal such as TGF-β1 on protein levels in a situation where there is no antibody available to detect the protein and the protein levels are too low to be detected by proteomics.

There exist DDX3 genes on the X (DDX3X) and Y (DDX3Y) chromosome (35). DDX3X escapes X chromosome inactivation, so females express 2 alleles, while males can express only one. DDX3X germline knockout leads to embryonic lethality (36, 37). The maternally derived DDX3X mutant allele (DDX3X^–/+^) results in 100% lethality for both male and female mice. Paternally derived DDX3X mutant allele (DDX3X^+/–^) is lethal for male mice, but female mice are born but have low body weights, have delayed development, and show signs of aspiration pneumonitis (36, 38). In humans, DDX3Y deletions cause subfertility or infertility (39). In mice, DDX3Y-knockout males show normal spermatogenesis, produce morphologically normal spermatozoa, and sire healthy offspring, suggesting that DDX3X may compensate for loss of DDX3Y (37).

In the bleomycin model of pulmonary fibrosis, treatment with the DDX3 inhibitor RK-33 potentiated survival, attenuated inflammation and fibrosis, and reduced lung tissue levels of DDX3, TGF-β1, and NEU3 in young male mice. DDX3 was also increased in human fibrotic lung tissue, suggesting that high levels of DDX3 may be associated with disease progression in IPF. Increased levels of DDX3 correlate with poor prognosis in many tumors, and treatment with RK-33 reduces tumor growth and improves survival (27, 40, 41). Possibly as a result of regulating RNA translation, DDX3 also inhibits apoptosis and promotes cell proliferation, adhesion, and motility, all factors that also promote lung inflammation and fibrosis.

In this paper and our previous work, we report a TGF-β1/DDX3/NEU3 /TGF-β1 positive feedback loop and show that inhibiting either DDX3 or NEU3 reduces lung fibrosis in bleomycin-treated mice. These results give us a better understanding of the effect of TGF-β1 on translation regulation in lung fibrosis and allow us to explore more specific TGF-β1–related therapeutic targets in inflammation and fibrosis.

## Methods

### Cell culture and TGF-β1 treatment.

Four HLF lines, from 2 healthy males (M2 and M3), 1 healthy female (F2), and 1 female IPF patient (F5), were gifts from Carol Feghali-Bostwick, Medical University of South Carolina, Charleston, South Carolina, USA. Cells were cultured in DMEM (Lonza) supplemented with 10% bovine calf serum (BCS) (Seradigm), 100 U/mL penicillin, 100 μg/mL streptomycin, and 2 mM glutamine (all from Lonza). For all experiments, cells were used at passages 4–8. HLFs (5 × 10^5^) were cultured in 100 mm tissue culture dishes (Corning) for 24–48 hours. When the cell density reached approximately 60% confluence, the supernatant was removed and cells were gently rinsed with prewarmed PBS 3 times. The medium was changed to 10 mL of prewarmed DMEM supplemented with 100 U/mL penicillin, 100 μg/mL streptomycin, and 2 mM glutamine (no BCS) with or without 10 ng/mL TGF-β1 (catalog 100-21C, PeproTech). After 48 hours, the cell density reached 80%–90% confluence, and cells were rinsed with 10 mL PBS 3 times before detaching with 1 mL Accutase (Innovative Cell Technologies) for 5 minutes. Cells were collected, then counted, and equal numbers of cells were lysed for the following assays.

### Proteomics.

HLFs (2 × 10^6^) were collected by centrifugation at 300*g* for 5 minutes at 4°C. The supernatant was discarded and cells were washed twice with 1 mL ice-cold PBS. The cell pellet was resuspended in 0.3 mL of ice-cold RIPA buffer (Thermo Fisher Scientific) with 1× Protease and Phosphatase Inhibitor (PPI) Cocktail (Thermo Fisher Scientific). Aliquots of the lysate were then snap-frozen in liquid nitrogen and stored at –80°C. In-gel protein preparation was performed as described on the University of Texas Southwestern Proteomics Core website (https://proteomics.swmed.edu/wordpress/?page_id=553). Samples were sent on ice to the University of Texas Southwestern Proteomics Core for trypsin digestion and Thermo Fisher Scientific Fusion Lumos standard gradient mass spectrometry. Briefly, protein gel fragments were reduced and alkylated with DTT (20 mM) and iodoacetamide (27.5 mM). A 0.1 μg/μL solution of trypsin in 50 mM triethylammonium bicarbonate was added to completely cover the gel. The resulting peptides were reconstituted in 10 μL of 2% (v/v) acetonitrile and 0.1% trifluoroacetic acid in water. A total of 5 μL of this solution was injected onto an Orbitrap Fusion Lumos mass spectrometer. The mass spectrometer operated in positive ion mode with a source voltage of 2.2 kV and an ion transfer tube temperature of 275°C. MS scans were acquired at 120,000 resolution in the Orbitrap, and up to 10 MS/MS spectra were obtained in the ion trap for each full spectrum acquired using higher energy collisional dissociation for ions with charges 2–7. Dynamic exclusion was set for 25 seconds after an ion was selected for fragmentation. Raw MS data files were analyzed using Proteome Discoverer v2.2 (Thermo Fisher Scientific), with peptide identification performed using Sequest HT searching against the human protein database from UniProt. The false discovery rate (FDR) cutoff was 1% for all peptides.

### Ribosomal RNA collection, purification, and sequencing.

HLFs (10 × 10^6^ to 20 × 10^6^) were collected and treated as described previously for monosome and polysome fractionation (14). Lysed samples were separated on a 10%–50% sucrose gradient made with polysome gradient buffer [10 mM HEPES-KOH pH 7.5, 70 mM NH_4_OAc, 5 mM Mg(OAc)_2_] and the associated (*w/v*) sucrose solutions prepared the same day. Cell lysates were layered on top of the prepared sucrose gradient, centrifuged at 288,000*g* for 2 hours at 4°C, and fractionated following the manufacturer’s instructions for a TRiAX flow cell (BioComp Instruments) and FC203B fraction collector (Gilson). RNA purification and precipitation was performed as described (14). RNA-Seq libraries were created following the manufacturer’s instructions using QuantSeq 3′ mRNA-Seq FWD library prep kits for Illumina (Lexogen), with 2 μg of RNA used as the starting material. Libraries were sequenced using an Illumina NextSeq 500 platform (Texas A&M University Institute for Genome Sciences and Society). RNA-sequencing data were analyzed using the QuantSeq Data Analysis Pipeline on the BlueBee Genomic Platform (Supplemental Data 2 for total RNA, Supplemental Data 3 for monosome and polysome mRNA). Briefly, the quality of sequences was evaluated using FastQC software (version 0.11.5) after adapter trimming with bbduk software (version 35.92). Gene and transcript intensities were computed using STAR software (version 2.5.2a) with Gencode Release 27 (GRCh38) as a reference. Differential expression analysis for mRNA was performed using R package DESeq2 (42).

### Proteomics and genomics data analysis.

For each protein or RNA, reads from the control (*n* = 4) and TGF-β1 group (*n* = 4) were compared by 2-tailed *t* test. For each significantly changed protein or RNA (*P* < 0.05), the TGF-β1/control ratio of the means was calculated. Proteins (Supplemental Data 5) or RNAs with a ratio > 1 were defined as “up” (statistically increased in the TGF-β1 group), and proteins (Supplemental Data 7) or RNAs with a ratio < 1 were defined as “down” (statistically decreased in TGF-β1 group). These ratios are linear fold-changes. For the proteins or RNAs that were not significantly changed, the 95% CI of the reads were compared between control and TGF-β1 groups using a standard statistical method (43, 44). Briefly, if the 95% CI of a protein or RNA in TGF-β1 group was completely within the range defined as 130% of the high end of the control CI and 70% of the low end of the control CI, the reads from this protein or RNA were considered statistically equivalent, and this protein (Supplemental Data 6) or RNA was then defined as “stable.” The nonchanged and nonstable proteins or RNAs were defined as “others.”

To determine the translation changes induced by monosome/polysome mRNA shifts, for the ribosomal mRNAs (*n* = 4), a PM ratio for each mRNA was calculated and compared between control and TGF-β1 groups with a *t* test. The higher the PM ratio is, the more mRNA will be translated. For the PM ratio significantly changed mRNAs (*P* < 0.05), an X value was calculated defined as: X = PM ratio in the presence of TGF-β1/PM ratio in control. mRNAs with X > 1 were defined as “M to P shifted” (PM ratio significantly increased in TGF-β1 group), and mRNAs with X < 1 were defined as “P to M shifted” (PM ratio significantly decreased in TGF-β1 group). For the mRNAs that did not show a PM ratio change, the 95% CI of the PM ratios were compared. If the 95% CI of an mRNA PM ratio in the TGF-β1 group was completely within the range defined as 130% of the high end of the control PM ratio CI and 70% of the low end of the control PM ratio CI for that mRNA, the PM ratios for this mRNA were considered statistically equivalent, and this protein was defined as “M/P stable.” The nonshifted and nonstable mRNAs were defined as “others.”

### GO analysis.

GO biological process analysis was performed using GO Panther (v17.0) (45), and analysis was confirmed and graphs generated by ShinyGO (v 0.74 using Ensembl 92 Release 104) (46). Groups were analyzed compared with the standard “all proteins” in the *Homo sapiens* database, and significance (*P* < 0.05) was determined by Fisher’s exact test with FDR correction.

### Common motif and uORF analysis.

mRNA sequences with 5′UTR, CDS, and 3′UTR were downloaded from the UCSC Genome Browser (http://genome.ucsc.edu). Common motifs of the mRNAs were identified using MEME (16). Briefly, mRNA sequences were uploaded to MEME as input, and common motifs were searched for using the “zoops” parameter and 10- to 25-nucleotide motifs. uORF analysis was performed at National Center for Biotechnology Information Open Reading Frame Finder (https://www.ncbi.nlm.nih.gov/orffinder/) with “ATG only” and “ATG and alternative initiation codons” settings.

### Plasmid construction, transfection, and quantitative PCR.

The human Myc-NEU3 expression plasmid RC216537 (Origene) was used to express NEU3. Myc-NEU3 mutants were generated using a QuikChange II Site-Directed Mutagenesis Kit (Agilent) with the DNA oligomers GGGCCCCTTAAACCACTTATTGAATCCACACTACC for mutant 1 and CAGTTCACTTAGACTGGAAGATGAATCTGGAACAC for mutant 2, (Supplemental Data 8), and the resulting plasmids were sequenced to confirm the point mutations and absence of other mutations. Human Myc-ESD and Myc-MKKS expression plasmids were constructed at VectorBuilder (Supplemental Data 8).

HLFs (1 × 10^5^) were mixed with 2 μg of 100 μg/mL of the above expression plasmids in 100 μL PBS (GE Lifesciences) and were transfected by electroporation using a 4D-Nucleofector System (Lonza) following the manufacturer’s protocol CZ-167. The transfected cells were kept at room temperature for 15 minutes for recovery, after which the cells were cultured in 10 mL DMEM with 10% BCS and 2 mM glutamine in a humidified incubator at 37°C with 5% CO_2_. After 24 hours, 400 μg/mL of G418 (Calbiochem EMD) was added to select for transfected cells. The culture medium with G418 was refreshed every 3 or 4 days. After 14 days, the cells were collected and used for assays described below. RNA extraction from the transfected cells and quantitative PCR for Myc-NEU3, Myc-ESD, and Myc-MKKS were done as we described previously (14) using the primers described in Supplemental Data 8.

### RBP prediction and RNA-protein interaction assay.

RBPs that can interact with group 4 mRNAs were identified using CLIP-Seq databases on POSTAR3 (http://111.198.139.65/RBS.html) with an RBP Binding Sites Module. RNA names of group 4 mRNAs were entered, and interacting RBPs were discovered from HIT-CLIP and iCLIP databases (18, 19, 47). The interaction probabilities of the group 4 motif GGAGGAGGAGGAGGAGGAGG and RBPs was predicted using RPISeq (http://pridb.gdcb.iastate.edu/RPISeq/#). For this analysis, the sequence of the motif and the sequence of each possible RBP were entered, and the interaction probability was predicted by a Random Forest classifier and a Support Vector Machine classifier (20, 48).

The protocol of the RNA-protein interaction assay followed previous reports (49, 50). Biotin-labeled group 4 motif (Bio-motif; Supplemental Data 8) was synthesized at IDT. A 50% slurry (5 μL) of streptavidin agarose beads (MilliporeSigma) was gently mixed with 500 μL ice-cold streptavidin agarose wash buffer (SWB) (20 mM Tris-HCl pH 7.5, 100 mM KCl, 2 mM EDTA, 0.5 mM DTT, 0.5 mM PMSF) in a 1.5 mL Eppendorf tube. The beads were collected by centrifugation at 500*g* for 2 minutes at 4°C, then resuspended in 500 μL ice-cold SWB, and 1 μL (1,000 pmol) of Bio-Motif was added to the tube and incubated with the beads at 4°C for 2 hours with 100 rpm rotation. The beads were collected by centrifugation at 500*g* for 2 minutes at 4°C. Beads were washed 3 times by resuspension in 500 μL Bio-Motif streptavidin interaction buffer (SIB) (20 mM Tris-HCl pH 7.5, 300 mM KCl, 0.2 mM EDTA, 0.5 mM DTT, 0.5 mM PMSF) followed by centrifugation. Beads were then resuspended in 500 μL SIB after the last rinse. Approximately 1 × 10^6^ cells treated with or without TGF-β1 as above were lysed with 200 μL lysis buffer (50 mM Tris-HCl pH 7.5, 150 mM NaCl, 5 mM EDTA, 1% NP-40, 1× PPI Cocktail) at 4°C for 30 minutes, and the mixture was clarified by centrifugation at 12,000*g* for 10 minutes at 4°C. The supernatant (100 μL, protein concentration at 3 μg/μL) was added to the beads and incubated at 4°C overnight with 100 rpm rotation. The beads were then collected by centrifugation at 500*g* for 2 minutes at 4°C and washed 3 times as described above with 400 μL SIB. Beads were resuspended in 40 μL SIB, 10 μL of 5× SDS sample buffer was added, and the mixture was heated at 95°C for 10 minutes. After cooling, this was clarified by centrifugation at 10,000*g* for 10 minutes at room temperature. The supernatant was collected and used for Coomassie blue staining and Western blotting.

### DDX3 siRNA transfection and DDX3 inhibitor treatment in HLFs.

HLFs were transfected with DDX3 siRNA (4392420, Thermo Fisher Scientific) or negative control siRNA (4390843, Thermo Fisher Scientific), following the manufacturer’s instructions. After 24 hours, the transfection medium was removed, and HLFs were treated with or without 10 ng/mL TGF-β1 in no serum–added DMEM. After an additional 48 hours, the culture medium was discarded, and cells were lysed in RIPA buffer with PPI cocktail. Samples were analyzed by Coomassie blue staining and Western blotting. Additionally, HLFs were treated with 1 or 10 μM RK-33 (Selleck Chemicals), or 1 or 10 μM IN-1 (AdooQ) from a 10 mM stock in DMSO (VWR) or DMSO diluent control, in the presence or absence of 10 ng/mL TGF-β1. After an additional 48 hours, the culture medium was discarded, and cells were lysed in RIPA buffer with PPI cocktail. Samples were analyzed by Coomassie blue staining and Western blotting.

### Pulmonary fibrosis mouse model and RK-33 treatment.

To induce inflammation and fibrosis, 7- to 8-week-old, 20–25 g, male C57BL/6 mice (Jackson Laboratories) were given an oropharyngeal aspiration of 4.5 U/kg bleomycin (Enzo Life Sciences) in 50 μL of 0.9% saline or oropharyngeal saline alone, as a control, as previously described (7, 8). All the mice were monitored twice daily to observe any sign of distress. At days 3, 7, 10, 14, and 21 after bleomycin treatment, mice were euthanized by CO_2_ inhalation. BALF was obtained and BALF cells counted, as described previously (7, 8). After collecting BALF, the lungs were inflated with and embedded in OCT compound (Leica), frozen, and stored at −80°C. Cryosections (10 μm) were cut on a CM1520 cryostat (Leica Biosystems) and used for staining as described below.

For mice receiving RK-33 treatment, inflammation and fibrosis were induced as described above. At days 10, 12, 14, 16, 18, and 20 after saline or bleomycin treatment, mice were given intraperitoneal injections of 20 mg/kg RK-33 (formulated as 40 mg/mL in DMSO) diluted in saline, or 100 μL DMSO/saline diluent control. Mice were euthanized at day 21 after bleomycin treatment. BALF and lung tissue were obtained and analyzed as described above. After euthanasia, the heart, kidneys, liver, spleen, interscapular brown adipose tissue, and epididymal white adipose tissue were collected and weighed.

### BALF cell counting.

The BALF cells were clarified and collected by centrifugation at 500*g* for 10 minutes at 4°C, and total BALF cell numbers were counted. BALF cell spots were prepared and stained with Wright-Giemsa stain (Polysciences) as previously described (7). Using a 40× objective, at least 150 cells from each stained BALF spot were examined, and the percentage positive cells was recorded.

### Picrosirius red stain and hydroxyproline assay.

Lung sections were stained with Picrosirius red to detect collagen and analyzed as previously described (7). Three randomly chosen 0.83 mm × 0.7 mm areas of each mouse lung section were used for the quantification of Picrosirius red staining (PolySciences) area. Hydroxyproline assays were performed as described previously (7, 9). Briefly, approximately half of lobes of lungs frozen in OCT were cut off in 3 pieces, thawed, and washed 3 times with 1 mL PBS to remove OCT by centrifugation at 2,000*g* for 5 minutes at room temperature in preweighed Eppendorf tubes. After the last centrifugation, the tubes were kept inverted for 5 minutes to allow PBS to blot onto blotting paper, and the tissue was then weighed. Tissues were then processed using a hydroxyproline quantification kit (MAK008-1KT, MilliporeSigma) following the manufacturer’s directions.

### Immunofluorescence and immunohistochemistry.

The Myc-NEU3–, Myc-ESD–, and Myc-MKKS–transfected and G418-selected HLFs were seeded on 96-well, black μ-Plates (catalog 89626, ibidi USA) at 2,000 cells per well. Cells were treated with or without 10 ng/mL TGF-β1 for 48 hours at 37°C in a humidified 5% CO_2_ incubator. Cells were fixed with 4% paraformaldehyde in PBS for 10 minutes at room temperature as previously described (51). Cells were rinsed with PBS 3 times and permeabilized with 0.5% Triton X-100 in PBS for 5 minutes. After 3 PBS rinses, cells were blocked with 2% IgG-free BSA (BSA-50, Rockland Immunochemicals) in PBS (PBSB) for 1 hour at room temperature. A total of 5 μg/mL rabbit anti-Myc-Tag antibody (catalog 2278, Cell Signaling Technology) in PBSB was incubated with cells overnight at 4°C. Cells were washed 3 times for 5 minutes per wash with PBS and stained with 1 μg/mL DAPI dye (BioLegend) and 2 μg/mL donkey F(ab′)_2_ anti-rabbit Alexa Fluor 488 (catalog 711-546-152, Jackson ImmunoResearch) or 5 μg/mL donkey F(ab′)_2_ anti-rabbit Rhodamine Red-X antibody (catalog 711-296-152, Jackson ImmunoResearch) in PBSB at room temperature for 30 minutes. Cells were washed 3 times with PBS, and images were taken with an Eclipse Ti2 microscope (Nikon).

Cryosections of mouse lungs were fixed in acetone for 20 minutes at room temperature and then rehydrated in water for 5 minutes and then PBS for 5 minutes. Slides were blocked with PBSB for 30 minutes and stained with 5 μg/mL rabbit antibodies in PBSB at 4°C overnight. Rabbit antibodies were anti–TGF-β1 (NBP1-45891), anti-GSTO1 (NBP2-32691), anti-IGBP1 (NBP1-83126), anti-MAGI3 (NBP1-81266), anti-MTCL1 (NBP2-47366), anti-LHFPL2 (NBP1-93654), anti-RBM33 (NBP1-84200), anti-BAF180 (NB100-79833), anti-APC4 (NBP1-90137) (all from Novus Biologicals), or anti-NEU3 (27879-1-AP, Proteintech). Monoclonal antibodies at 5 μg/mL were rat anti-CD45 (catalog 304002, BioLegend), rat anti-CD11b (catalog 101202, BioLegend), and hamster anti-CD11c (catalog 117301, BioLegend). Directly conjugated antibodies at 5 μg/mL were Alexa Fluor 647–conjugated anti-CD45 (catalog 103123, BioLegend), Alexa Fluor 488–conjugated anti-TGF-β1 (NBP2-45137AF488, Novus Biologicals), and Alexa Fluor 647–conjugated anti-DDX3 (NBP2-14848AF647, Novus Biologicals). The staining was further processed as described previously (7, 52). Briefly, slides were incubated with combinations of 2 μg/mL donkey F(ab′)_2_ anti-rabbit Alexa Fluor 488 (Jackson ImmunoResearch), 2 μg/mL goat F(ab′)_2_ anti-hamster Alexa Fluor 488 (catalog 107-546-142, Jackson ImmunoResearch), 5 μg/mL donkey F(ab′)_2_ anti-rat Rhodamine Red-X antibody (catalog 712-296-153 Jackson ImmunoResearch), and 5 μg/mL donkey F(ab′)_2_ anti-rabbit Rhodamine Red-X antibody (Jackson ImmunoResearch) in PBSB at room temperature for 30 minutes. The staining was further processed as described previously (7, 52). The slides were kept in the dark at 4°C for 1 hour to harden the antifade mounting media with DAPI (catalog H-1500, Vector Laboratories).

Human lung tissue sections were obtained from the NIH National Heart Lung and Blood Institute–sponsored Lung Tissue Research Consortium. Detailed information can be found in Supplemental Table 3. Formalin-fixed, paraffin-embedded slides were dewaxed with xylene, then rehydrated through a graded series of alcohols, distilled water, and then PBS (53). Slides were then blocked by incubation in PBSB for 60 minutes. Endogenous biotin was blocked by the addition of streptavidin and biotin solutions, following the manufacturer’s instructions (Streptavidin/Biotin Blocking Kit, Vector Laboratories). Slides were then incubated overnight at 4°C with 1 μg/mL rabbit anti-DDX3 (NBP1-85291, Novus Biologicals) in PBSB. After washing, primary antibodies were detected with 1 μg/mL biotinylated donkey F(ab′)_2_ anti-rabbit IgG (711-066-152; Jackson ImmunoResearch) in PBSB for 30 minutes. Biotinylated antibodies were detected by a 1:500 dilution of ExtrAvidin alkaline phosphatase (Vector Laboratories) in PBSB for 30 minutes. Staining was developed with the Vector Red Alkaline Phosphatase Kit (Vector Laboratories) for 10 minutes. Sections were then counterstained for 30 seconds with Gill’s hematoxylin 3 (MilliporeSigma). Slides were mounted with VectaMount (Vector Laboratories). Tissue sections stained with antibodies were imaged with an Eclipse Ti2 microscope and analyzed with ImageJ2 software (NIH). The percentage area of tissue stained with was quantified as a percentage of the total area of the tissue, as described previously (6, 7).

### Coomassie blue staining and Western blotting.

Approximately 10^6^ cells were collected by centrifugation as described above and lysed in 200 μL RIPA buffer (Thermo Fisher Scientific) with 1× PPI Cocktail (Thermo Fisher Scientific). A total of 80 μL of cell lysate was mixed with 20 μL 5× SDS protein loading buffer and heated at 95°C for 5 minutes. Equal volumes of the heated samples were loaded on 4%–20% Tris–glycine Mini-Protean TGX gels (Bio-Rad) for SDS-PAGE. The gel was stained with freshly prepared Coomassie blue dye (Thermo Fisher Scientific) for 1 hour with gentle shaking and destained overnight. Western blots were done as previously described (14), staining with 0.5 μg/mL rabbit anti–Myc-Tag antibody (Cell Signaling Technology). Where indicated, blots were stained with 1 μg/mL rabbit antibodies against AGO2 (NBP2-67121), UPF1 (NBP1-89641), DDX3 (NBP1-85291), U2AF2 (NBP2-58989), CELF2 (NBP2-16035), ALYREF (NBP1-90179), GSTO1 (NBP2-32691), IGBP1 (NBP1-83126), MAGI3 (NBP1-81266), LHFPL1 (NBP2-47366), RBM33 (NBP1-84200), BAF180 (NB100-79833) (all from Novus Biologicals), phospho-AGO2 (AP5291, ECM Biosciences), phospho-UPF1 (07-1016, MilliporeSigma), or NEU3 (27879-1-AP, Proteintech). Peroxidase-conjugated donkey F(ab′)_2_ anti-rabbit (catalog 711-036-152, Jackson ImmunoResearch) was used as the secondary antibody. SuperSignal West Pico Chemiluminescence Substrate (Thermo Fisher Scientific) was used following the manufacturer’s protocol to visualize the peroxidase using a ChemiDoc XRS+ System (Bio-Rad).

### Raw data accessibility.

Raw files of proteomics data were uploaded to MassIVE (data set MSV000089217, https://massive.ucsd.edu/ProteoSAFe/dataset.jsp?task=dd7c4a6aac1145d1bc6ac9ead122f015

Raw files of RNA-Seq data were uploaded to GEO (accession number GSE201506).

### Statistics.

Statistical analysis was performed using Prism v7 (GraphPad Software). Statistical significance between 2 groups was determined by 2-tailed *t* test, or between multiple groups using 1-way ANOVA with Dunnett’s posttest, and significance was defined as *P* < 0.05. All P values were adjusted and verified with Bonferroni correction. For the multiple *t* tests in the proteomics and RNA-Seq data analysis, an FDR approach was used with *q* = 0.05. Statistical equivalence between 2 groups was determined by the 95% CIs.

### Study approval.

This study was carried out in strict accordance with the recommendations in the *Guide for the Care and Use of Laboratory Animals* of the NIH (National Academies Press, 2011). The protocol was approved by Texas A&M University Animal Use and Care Committee (IACUC 2020-0272). All procedures were performed under anesthesia, and all efforts were made to minimize suffering. Human cells and tissue samples were deidentified, and the protocol was approved by the Texas A&M University Institutional Review Board.

## Author contributions

WC and RHG conceived the study; WC and RHG designed methodology; WC and DP investigated; WC, DP, and RHG wrote the original draft; WC, DP, and RHG reviewed and edited the draft; and RHG acquired funding.

## Supplementary Material

Supplemental data

Supplemental data set 1

Supplemental data set 2

Supplemental data set 3

Supplemental data set 4

Supplemental data set 5

Supplemental data set 6

Supplemental data set 7

Supplemental data set 8

## Version 1 02/23/2023

In-Press Preview

## Version 2 04/10/2023

Electronic publication
