# Supplementary material for: The mRNA-binding protein DDX3 mediates TGF-β1 upregulation of translation and promotes pulmonary fibrosis
Source: JCI Insight. 2023 Apr 10;8(7):e167566. doi: 10.1172/jci.insight.167566 (PMC10132153; doi:10.1172/jci.insight.167566)
Supplement: Supplemental data [file jciinsight-8-167566-s082.pdf]

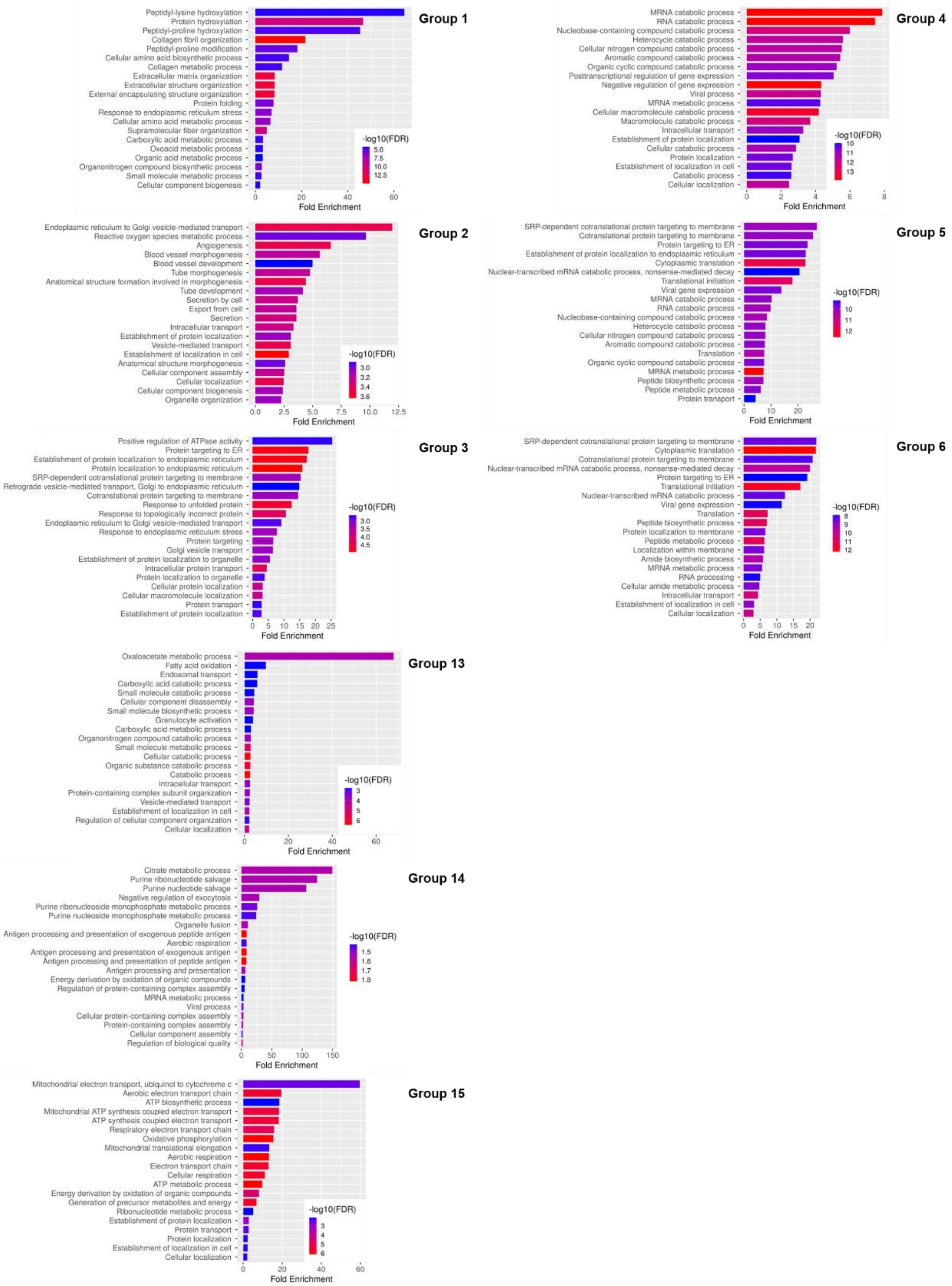

Figure S1. Gene ontology (GO) biological process analysis of groups with 20 or more proteins. Significance ($p < 0.05$) was determined by Fisher's exact test with False Discovery Rate (FDR) correction.

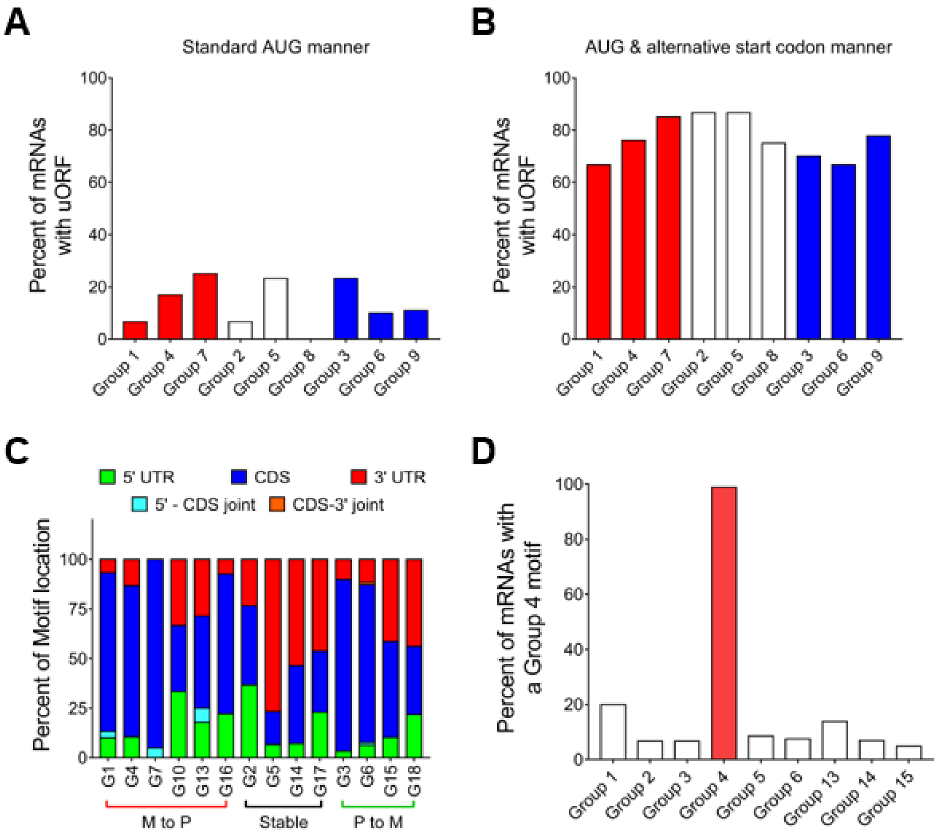

Figure S2. Percent of mRNAs with an upstream open reading frame (uORF), locations of motifs in mRNAs and occurrence of Group 4 motif in other groups. (A-B) Each mRNA in the indicated group was assessed for an uORF with ether an AUG near the 5' end of the ORF or an AUG or alternative start codon near the 5' end of the uORF. **(C)** Motif location was analyzed in mRNAs which showed a TGF-β1-induced monosome to polysome (M to P) shift (groups 1, 4, 7, 10, 13, 16), mRNAs which showed a polysome to monosome (P to M) shift (groups 3, 6, 15, 18), and mRNAs which showed no change with TGF-β1 treatment (stable). **(D)** The occurrence of Group 4 motifs was screened in mRNAs of groups with 20 or more proteins.

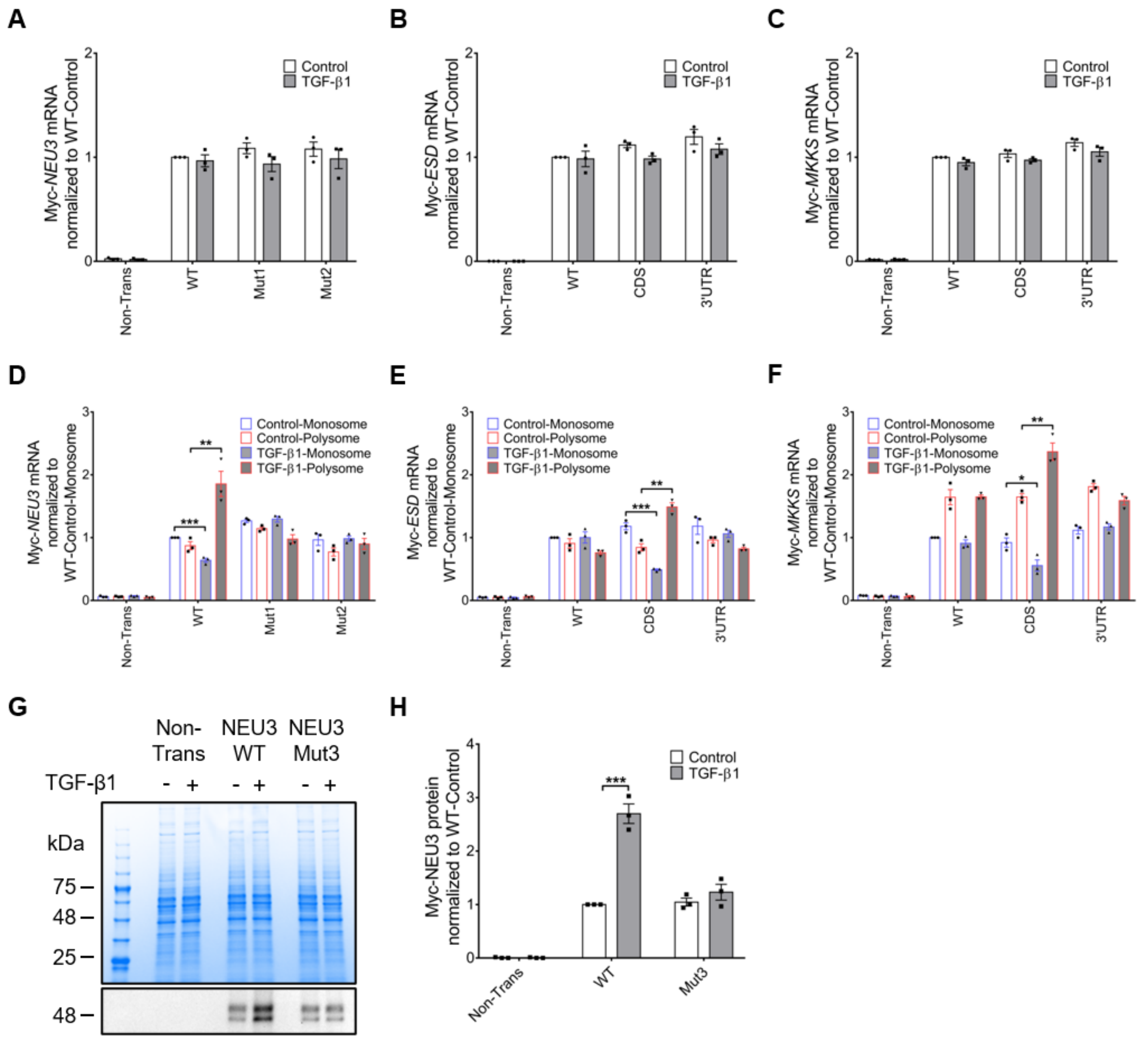

Figure S3. The group 4 motif is necessary and sufficient for TGF-β1-induced monosome/polysome mRNA shifts. (A-C) Human lung fibroblasts transfected with the indicated constructs were cultured without (control) or with TGF-β1. Total Myc-*NEU3*, Myc-*ESD*, and Myc-*MKKS* mRNA levels were determined by qPCR with primers targeting the junction between the Myc-tag and NEU3, ESD, or MKKS sequences. **(D-F)** Ribosomal Myc-*NEU3*, Myc-*ESD*, and Myc-*MKKS* mRNA levels were determined by qPCR with primers targeting the junction between

the Myc-tag and NEU3, ESD, or MKKS sequences. **(G)** Levels of Myc-NEU3 in transfected and non-transfected HLFs was assayed by western blotting with an anti-Myc-tag antibody. NEU3 WT is myc-tagged NEU3, and NEU3-Mut3 is myc-tagged NEU3 containing a mutation of the NEU3 coding region Group 4 motif where the mutation changes the nucleotide sequence of the motif without changing the encoded amino acids. Top image is a Coomassie stained gel of cell lysates, bottom image is western blot. Gel and western blot are representative of 3 independent experiments. **(H)** Quantification of **G**. Values in A-F and H are mean ± SEM, n=3. * $p < 0.05$, ** $p < 0.01$, *** $p < 0.001$ (t-tests).

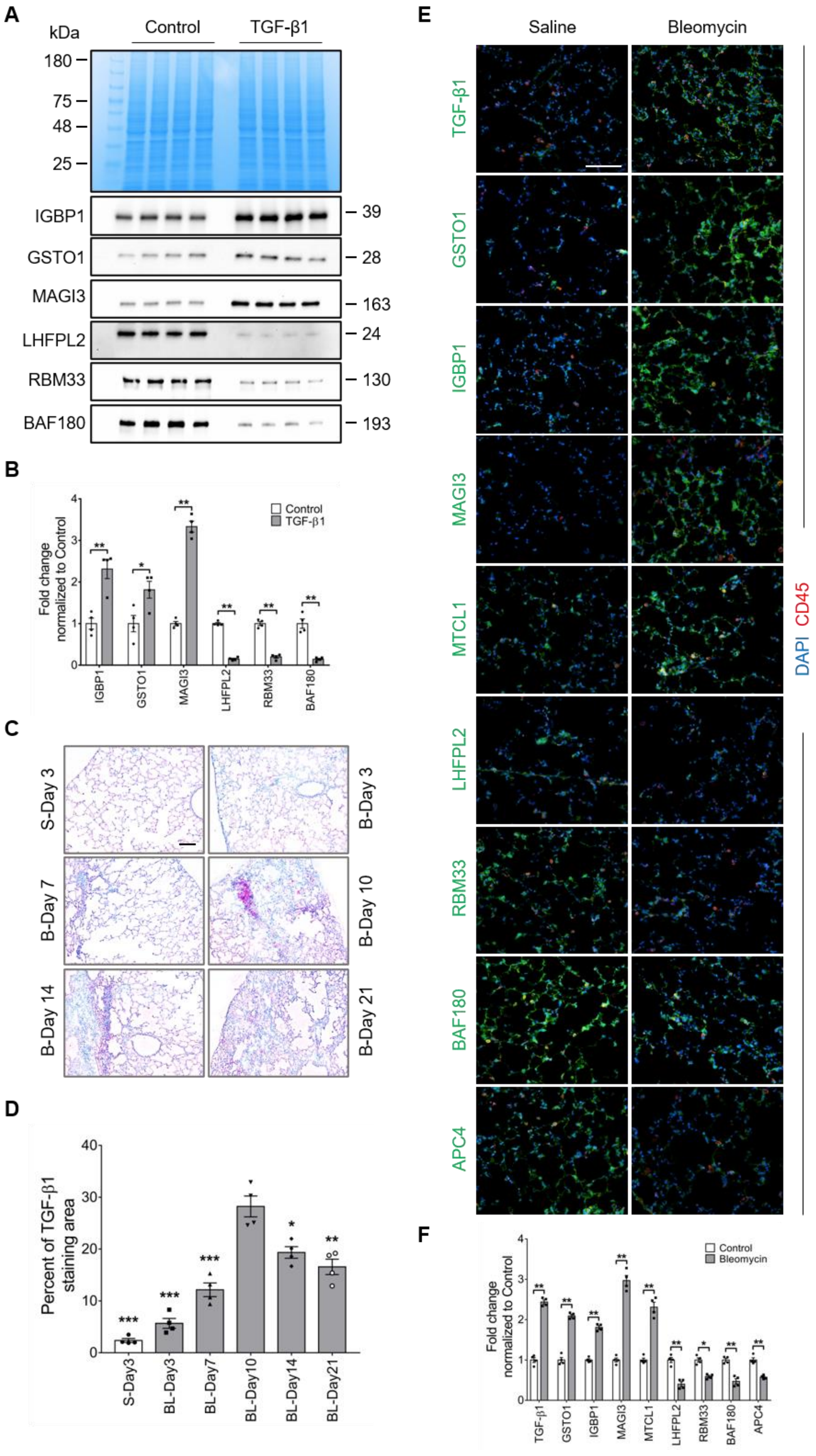

A
kDa
Control
TGF-β1
180
75
48
25
IGBP1
GSTO1
MAGI3
LHFPL2
RBM33
BAF180
39
28
163
24
130
193
B
Fold change normalized to Control
Control
TGF-β1
IGBP1
GSTO1
MAGI3
LHFPL2
RBM33
BAF180
C
S-Day 3
B-Day 3
B-Day 7
B-Day 10
B-Day 14
B-Day 21
D
Percent of TGF-β1 staining area
S-Day3
BL-Day3
BL-Day7
BL-Day10
BL-Day14
BL-Day21
E
Saline
Bleomycin
TGF-β1
GSTO1
IGBP1
MAGI3
MTCL1
LHFPL2
RBM33
BAF180
APC4
DAPI CD45
F
Fold change normalized to Control
Control
Bleomycin
TGF-β1
GSTO1
IGBP1
MAGI3
MTCL1
LHFPL2
RBM33
BAF180
APC4

Figure S4. Predictions verified in fibroblasts and mouse lungs. (A) Human lung fibroblasts were cultured without (control) or with TGF-β1 and then western blots of cell lysates were stained with anti-IGBP1, GSTO1, MAGI3, LHFPL2, RBM33 or BAF180 antibodies. Top image is Coomassie stained gel of cell lysates, bottom image is western blot. Molecular masses in kDa are at left for the Coomassie gel and at right for the western blots. Pairs of columns (control and TGF-β1) are independent experiments. **(B)** Quantification of **A**. Values are mean ± SEM, n = 4. * $p < 0.05$, ** $p < 0.01$ (t-tests). **(C)** Mouse lung tissues collected at day 3, 7, 10, 14 and 21 after saline (S) or bleomycin (BL) treatment were stained with anti-TGF-β1 antibodies. Red indicates TGF-β1 positive stain. Bar is 100 μm. Images are representative of 4 independent experiments (3 male mice and 1 female mouse). **(D)** Quantification of **C**. Values are mean ± SEM, n = 4. * $p < 0.05$, ** $p < 0.01$, *** $p < 0.001$ vs BL-Day 10 (1-way ANOVA, Dunnett's test). **(E)** Mouse lung tissues collected at day 10 after saline or bleomycin treatment were stained with anti-TGF-β1, GSTO1, IGBP1, MAGI3, MTCL1, LHFPL2, RBM33, BAF180, or APC4 antibodies and anti-CD45 antibodies. Red is CD45 positive stain, green is positive stain for the indicated protein, and blue is DAPI staining of nuclei. Bar is 100 μm. Images are representative of 4 independent experiments (3 male and 1 female mice). **(F)** quantification of **E**. Values are mean ± SEM, n = 4. * $p < 0.05$, ** $p < 0.01$ (t-tests).

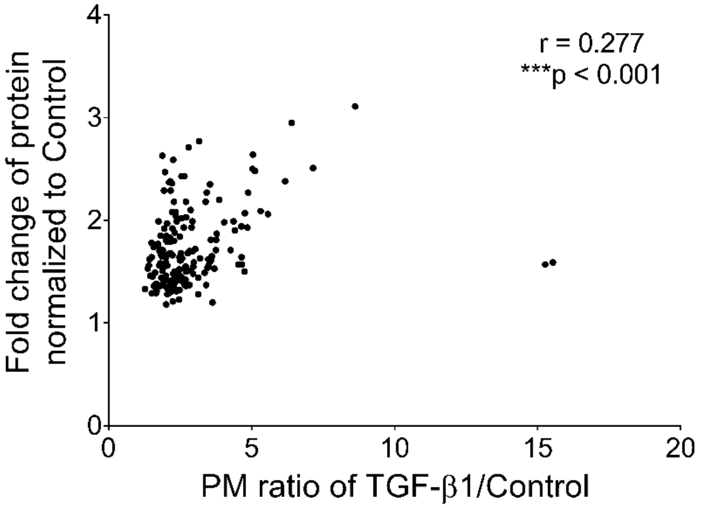

Figure S5. Correlation analysis of PM ratio and protein level change. The correlation between PM ratio and protein level change with or without TGF-β1 treatment was analyzed in 181 proteins of Group 4. *** $p < 0.001$ (Pearson's correlation test).

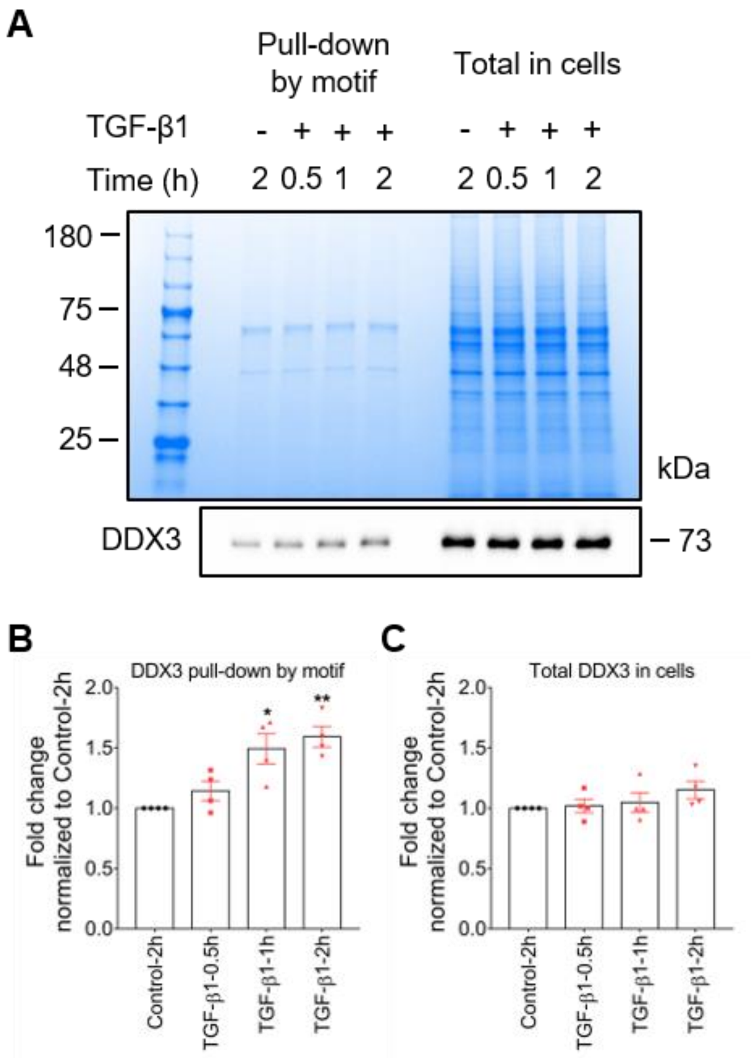

Figure S6. Group 4 motif and DDX3 binding in a short time points TGF-β1 treatment assay.

(A) The biotin-tagged RNA motif was used to pull down material from control and TGF-β1-treated cells. The cells were treated with or without TGF-β1 for 0.5, 1 and 2 hours. The Coomassie-stained gel shows the material in the pull-down samples and in the total cell lysates, and the Western blot shows staining of DDX3. Gel and western blot are representative of 4 independent experiments. **(B-C)** Quantification of DDX3 levels in pull-down samples and total samples. Values in **B** and **C** are mean ± SEM, n=4. * $p < 0.05$, ** $p < 0.01$ (1-way ANOVA, Dunnett's test).

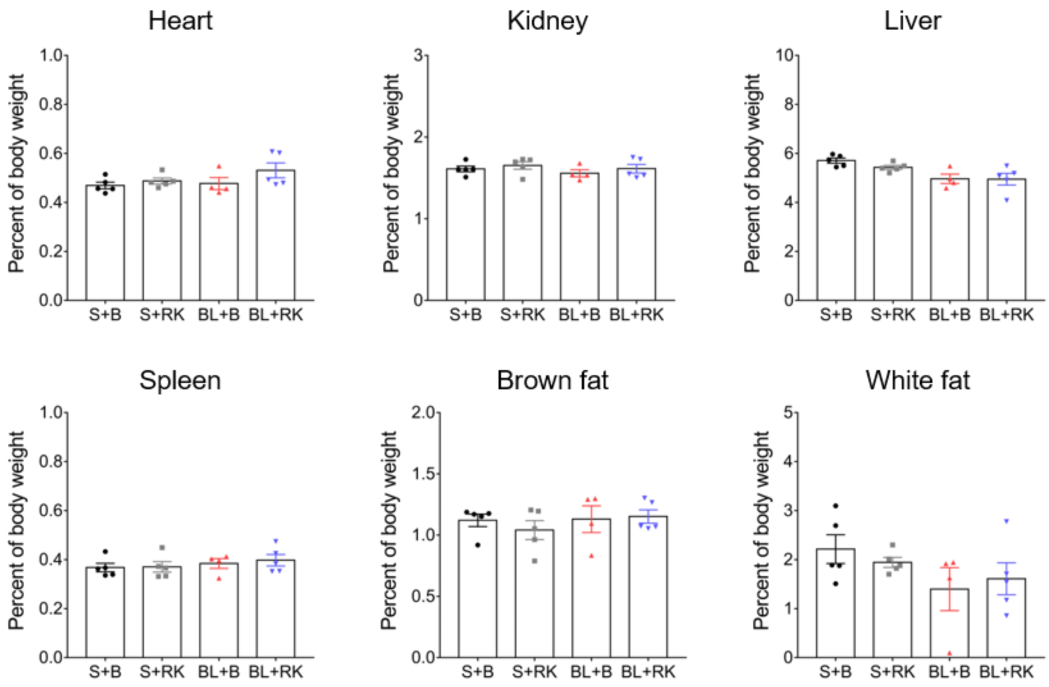

Figure S7. Injections of RK-33 had no significant effect on organ weights. Weights of heart, kidneys, liver, spleen, interscapular brown fat, and epididymal white fat as percentage of total body weight at day 21. Values are mean ± SEM, n = 5 mice for S+B, S+RK, and BL+RK, n = 4 mice for BL+B. For each tissue, there were no significant differences as determined by t test or 1-way ANOVA (Dunnett's test).

Table S1. Protein groups based on TGF-β1 effects on protein, total mRNA, and mRNA monosome/polysome levels

Group	Total Protein	Total mRNA	Ribosomal mRNA	Number of Proteins	Common Motif
1	↑	↑	M → P	156	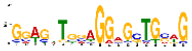
2	↑	↑	MP stable	68	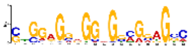
3	↑	↑	P → M	80	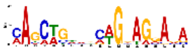
4	↑	Stable	M → P	181	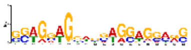
5	↑	Stable	MP stable	82	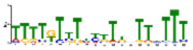
6	↑	Stable	P → M	93	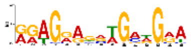
7	↑	↓	M → P	20	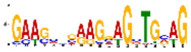
8	↑	↓	MP stable	4	N/A
9	↑	↓	P → M	9	N/A
10	Stable	↑	M → P	14	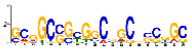
11	Stable	↑	MP stable	6	N/A
12	Stable	↑	P → M	13	N/A
13	Stable	Stable	M → P	144	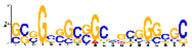
14	Stable	Stable	MP stable	58	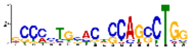
15	Stable	Stable	P → M	81	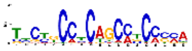
16	Stable	↓	M → P	52	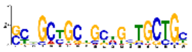
17	Stable	↓	MP stable	27	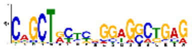
18	Stable	↓	P → M	34	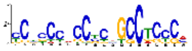
19	↓	↑	M → P	1	N/A
20	↓	↑	MP stable	0	N/A
21	↓	↑	P → M	0	N/A
22	↓	Stable	M → P	6	N/A
23	↓	Stable	MP stable	1	N/A
24	↓	Stable	P → M	2	N/A
25	↓	↓	M → P	5	N/A
26	↓	↓	MP stable	6	N/A
27	↓	↓	P → M	6	N/A

Grouping of proteins and their encoding mRNAs where there was a statistically significant TGF-β1-induced increase (upward arrow), absence of a change (Stable), or decrease (downward arrow) for the protein, the levels of the encoding mRNA, and the polysome/monosome ratio for the mRNA. Common motifs observed in the mRNAs for each group are indicated at right. N/A indicates that there was either no detectable common motif, or too few mRNAs in the group to identify a common motif. M indicates Monosome, P indicates Polysome.

Table S2. Summary of the results from Figure S4

ID	Group	Contain group 4 motif?	Protein	Total mRNA	Mono / Poly mRNA	Bleomycin treated mice	TGF-β1 treated HLF cells
IGBP1	7	Yes	↑	↓	M → P shift	↑	↑
GSTO1	7	No	↑	↓	M → P shift	↑	↑
MAGI3	N/A	Yes	No proteomics reads	Stable	M → P shift	↑	↑
MTCL1	N/A	Yes	No proteomics reads	Stable	M → P shift	↑	Ab N/A for WB
LHFPL2	24	No	↓	Stable	P → M shift	↓	↓
RBM33	24	No	↓	Stable	P → M shift	↓	↓
BAF180	N/A	No	No proteomics reads	Stable	P → M shift	↓	↓
APC4	N/A	No	No proteomics reads	Stable	P → M shift	↓	Ab N/A for WB

An upward arrow indicates a TGF-β1-induced or bleomycin-induced increase, a downward arrow indicates a decrease. Ab N/A for WB indicates the antibody was unable to detect the protein on western blots of lysates from human lung fibroblasts. M indicates Monosome, P indicates Polysome.

Table S3. Clinical data

Group	FVC (mean ± SD)	Sex	Age in years (mean ± SD)	Clinical Details
**ILD <50%**	34.25 ± 7.5	6 male<br>3 female	49.7 ± 11.7	n=6 UIP<br>n=1 COP<br>n=1 NSIP<br>n=1 fibrosis
**COPD >80%**	86.9 ± 6.8	6 male<br>5 female	67.0 ± 11.7	n=11<br>COPD/ Emphysema
**t-test (Mann-Whitney)**	p <0.0001	ns	p = 0.0043	

Clinical data from the National Heart Lung and Blood Institute-sponsored Lung Tissue Research Consortium (LTRC) sections used in Figures 3G and H. Pulmonary function test - Forced vital capacity (FVC). Clinical diagnosis: Chronic obstructive pulmonary disease (COPD); Fibrosis indicates uncharacterized interstitial lung disease (ILD); Usual interstitial pneumonia (UIP); Cryptogenic organizing pneumonia (COP); non-specific interstitial pneumonitis (NSIP).
