# Supplementary material for: The mRNA-binding protein DDX3 mediates TGF-β1 upregulation of translation and promotes pulmonary fibrosis
Source: JCI Insight. 2023 Apr 10;8(7):e167566. doi: 10.1172/jci.insight.167566 (PMC10132153; doi:10.1172/jci.insight.167566)
Supplement: Supplemental data set 8 [file jciinsight-8-167566-s090.pdf]

a. Myc-NEU3 plasmids and primers

1. Plasmid map of Myc-NEU3-WT:

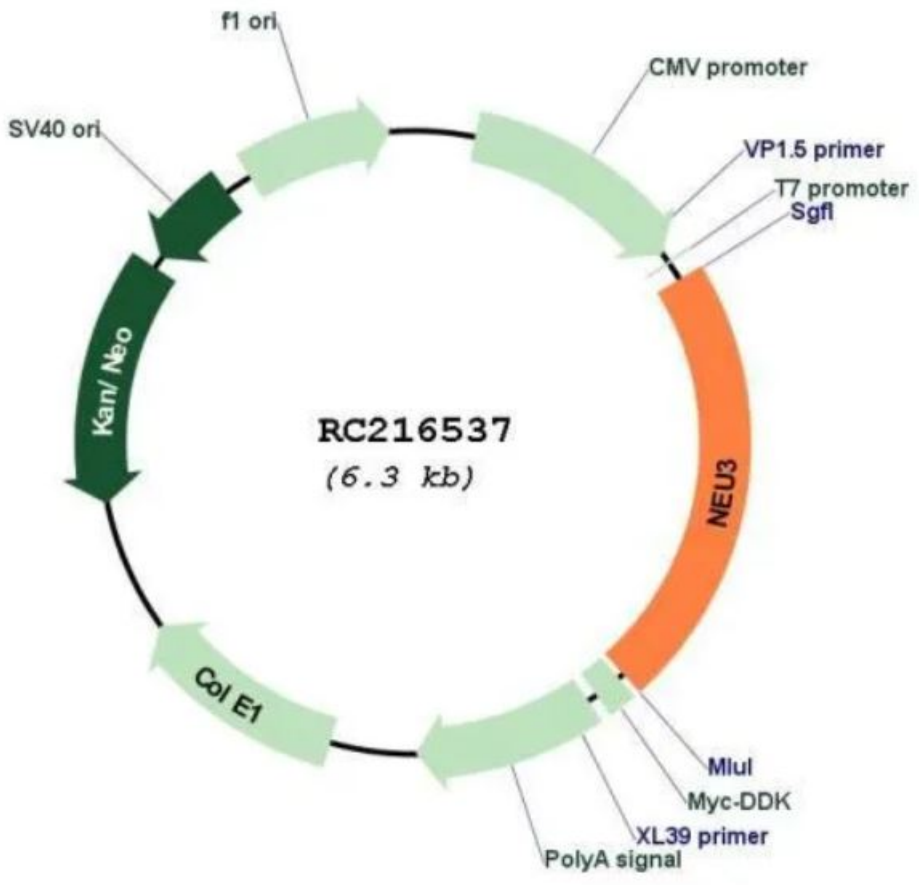

2. Nucleotide Sequences of Myc-NEU3-WT:

>RC216537 representing NM_006656
Red=Cloning site Blue=ORF Green=Tags(s)

TTTTGTAATACGACTCACTATAGGGCGGCCGGGAATTCGTCGACTGGATCCGGTACCGAGGAGAT
CTGCCGCCGCGATCGCCATGAGACCTGCGGACCTGCCCCCGCGCCCCATGGAAGAATCCCCGGCG
TCCAGCTCTGCCCCGACAGAGACGGAGGAGCCGGGGTCCAGTGCAGAGGTCATGGAAGAAGTGA
CAACATGCTCCTTCAACAGCCCTCTGTTCCGGCAGGAAGATGACAGAGGGATTACCTACCGGATC
CCAGCCCTGCTCTACATACCCCCCACCCACACCTTCCTGGCCTTTGCAGAGAAGCGTTCTACGAGG
AGAGATGAGGATGCTCTCCACCTGGTGCTGAGGCGAGGGTTGAGGATTGGGCAGTTGGTACAGTG
GGGGCCCCTGAAGCCACTGATGGAAGCCACACTACCGGGGCATCGGACCATGAACCCCTGTCCTG
TATGGGAGCAGAAGAGTGGTTGTGTGTTCCTGTTCTTCATCTGTGTGCGGGGCCATGTCACAGAGC
GTCAACAGATTGTGTCAGGCAGGAATGCTGCCCGCCTTTGCTTCATCTACAGTCAGGATGCTGGAT
GTTCATGGAGTGAGGTGAGGGACTTGACTGAGGAGGTCATTGGCTCAGAGCTGAAGCACTGGGCC
ACATTTGCTGTGGGCCCAGGTCATGGCATCCAGCTGCAGTCAGGGAGACTGGTCATCCCTGCGTAT
ACCTACTACATCCCTTCCTGGTTCTTTTGCTTCCAGCTACCATGTAAAACCAGGCCTCATTCTCTGA
TGATCTACAGTGATGACCTAGGGGTCACATGGCACCATGGTAGACTCATTAGGCCCATGGTTACA
GTAGAATGTGAAGTGGCAGAGGTGACTGGGAGGGCTGGCCACCCTGTGCTATATTGCAGTGCCCG

GACACCAAACAGGTGCCGGGCAGAGGCGCTCAGCACTGACCATGGTGAAGGCTTTCAGAGACTG
GCCCTGAGTCGACAGCTCTGTGAGCCCCCACATGGTTGCCAAGGGAGTGTGGTAAGTTTCCGGCC
CCTGGAGATCCCACATAGGTGCCAGGACTCTAGCAGCAAAGATGCACCCACCATTCAGCAGAGCT
CTCCAGGCAGTTCACTGAGGCTGGAGGAGGAAGCTGGAACACCGTCAGAATCATGGCTCTTGTAC
TCACACCCAACCAGTAGGAAACAGAGGGTTGACCTAGGTATCTATCTCAACCAGACCCCCTTGGA
GGCTGCCTGCTGGTCCCGCCCCTGGATCTTGCACTGTGGGCCCTGTGGCTACTCTGATCTGGCTGC
TCTGGAGGAGGAGGGCTTGTTTGGGTGTTTGTTTGAATGTGGGACCAAGCAAGAGTGTGAGCAGA
TTGCCTTCCGCCTGTTTACACACCGGGAGATCCTGAGTCACCTGCAGGGGGACTGCACCAGCCCTG
GTAGGAACCCAAGCCAATTCAAAAGCAATACGCGTACGCGGCCGCTC**GAGCAGAAACTCATCTC**
AGAAGAGGATCTGGCAGCAAATGATATCCTG**GATTACAAGGATGACGACGATAAG**GTTTAA

The two Group 4 motifs in NEU3 coding sequence are labeled with pink background.

3. Nucleotide Sequences of Myc-NEU3-Mutant1:

TTTTGTAATACGACTCACTATAGGGCGGCCGGGAATTCGTCGACTGGATCCGGTACCGAGGAGAT
CTGCCGCCGCGATCGCCATGAGACCTGCGGACCTGCCCCCGCGCCCCATGGAAGAATCCCCGGCG
TCCAGCTCTGCCCCGACAGAGACGGAGGAGCCGGGGTCCAGTGCAGAGGTCATGGAAGAAGTGA
CAACATGCTCCTTCAACAGCCCTCTGTTCCGGCAGGAAGATGACAGAGGGATTACCTACCGGATC
CCAGCCCTGCTCTACATACCCCCCACCCACACCTTCCTGGCCTTTGCAGAGAAGCGTTCTACGAGG
AGAGATGAGGATGCTCTCCACCTGGTGCTGAGGCGAGGGTTGAGGATTGGGCAGTTGGTACAGTG
GGGGCCCCTTAAACCACTTATTGAATCCACACTACCGGGGCATCGGACCATGAACCCCTGTCCTGT
ATGGGAGCAGAAGAGTGGTTGTGTGTTCCTGTTCTTCATCTGTGTGCGGGGCCATGTCACAGAGCG
TCAACAGATTGTGTCAGGCAGGAATGCTGCCCGCCTTTGCTTCATCTACAGTCAGGATGCTGGATG
TTCATGGAGTGAGGTGAGGGACTTGACTGAGGAGGTCATTGGCTCAGAGCTGAAGCACTGGGCCA
CATTTGCTGTGGGCCCAGGTCATGGCATCCAGCTGCAGTCAGGGAGACTGGTCATCCCTGCGTATA
CCTACTACATCCCTTCCTGGTTCTTTTGCTTCCAGCTACCATGTAAAACCAGGCCTCATTCTCTGAT
GATCTACAGTGATGACCTAGGGGTCACATGGCACCATGGTAGACTCATTAGGCCCATGGTTACAG
TAGAATGTGAAGTGGCAGAGGTGACTGGGAGGGCTGGCCACCCTGTGCTATATTGCAGTGCCCGG
ACACCAAACAGGTGCCGGGCAGAGGCGCTCAGCACTGACCATGGTGAAGGCTTTCAGAGACTGGC
CCTGAGTCGACAGCTCTGTGAGCCCCCACATGGTTGCCAAGGGAGTGTGGTAAGTTTCCGGCCCCT
GGAGATCCCACATAGGTGCCAGGACTCTAGCAGCAAAGATGCACCCACCATTCAGCAGAGCTCTC
CAGGCAGTTCACTGAGGCTGGAGGAGGAAGCTGGAACACCGTCAGAATCATGGCTCTTGTACTCA
CACCCAACCAGTAGGAAACAGAGGGTTGACCTAGGTATCTATCTCAACCAGACCCCCTTGGAGGC
TGCCTGCTGGTCCCGCCCCTGGATCTTGCACTGTGGGCCCTGTGGCTACTCTGATCTGGCTGCTCTG
GAGGAGGAGGGCTTGTTTGGGTGTTTGTTTGAATGTGGGACCAAGCAAGAGTGTGAGCAGATTGC
CTTCCGCCTGTTTACACACCGGGAGATCCTGAGTCACCTGCAGGGGGACTGCACCAGCCCTGGTA
GGAACCCAAGCCAATTCAAAAGCAATACGCGTACGCGGCCGCTC**GAGCAGAAACTCATCTCAGA**
AGAGGATCTGGCAGCAAATGATATCCTG**GATTACAAGGATGACGACGATAAG**GTTTAA

5 sites were mutated in the 1st Group 4 motif in NEU3 with the following mutation primers:

Forward GGGCCCCTTAAACCACTTATTGAATCCACACTACC

Reverse GGTAGTGTGGATTCAATAAGTGGTTTAAGGGGCCC

4. Nucleotide Sequences of Myc-NEU3-Mutant2:

TTTTGTAATACGACTCACTATAGGGCGGCCGGGAATTCGTCGACTGGATCCGGTACCGAGGAGAT
CTGCCGCCGCGATCGCCATGAGACCTGCGGACCTGCCCCCGCGCCCCATGGAAGAATCCCCGGCG
TCCAGCTCTGCCCCGACAGAGACGGAGGAGCCGGGGTCCAGTGCAGAGGTCATGGAAGAAGTGA
CAACATGCTCCTTCAACAGCCCTCTGTTCCGGCAGGAAGATGACAGAGGGATTACCTACCGGATC
CCAGCCCTGCTCTACATACCCCCCACCCACACCTTCCTGGCCTTTGCAGAGAAGCGTTCTACGAGG
AGAGATGAGGATGCTCTCCACCTGGTGCTGAGGCGAGGGTTGAGGATTGGGCAGTTGGTACAGTG
GGGGCCCCTGAAGCCACTGATGGAAGCCACACTACCGGGGCATCGGACCATGAACCCCTGTCCTG
TATGGGAGCAGAAGAGTGGTTGTGTGTTCCTGTTCTTCATCTGTGTGCGGGGCCATGTCACAGAGC
GTCAACAGATTGTGTCAGGCAGGAATGCTGCCCGCCTTTGCTTCATCTACAGTCAGGATGCTGGAT
GTTCATGGAGTGAGGTGAGGGACTTGACTGAGGAGGTCATTGGCTCAGAGCTGAAGCACTGGGCC
ACATTTGCTGTGGGCCCAGGTCATGGCATCCAGCTGCAGTCAGGGAGACTGGTCATCCCTGCGTAT
ACCTACTACATCCCTTCCTGGTTCTTTTGCTTCCAGCTACCATGTAAAACCAGGCCTCATTCTCTGA
TGATCTACAGTGATGACCTAGGGGTCACATGGCACCATGGTAGACTCATTAGGCCCATGGTTACA
GTAGAATGTGAAGTGGCAGAGGTGACTGGGAGGGCTGGCCACCCTGTGCTATATTGCAGTGCCCG
GACACCAAACAGGTGCCGGGCAGAGGCGCTCAGCACTGACCATGGTGAAGGCTTTCAGAGACTG
GCCCTGAGTCGACAGCTCTGTGAGCCCCCACATGGTTGCCAAGGGAGTGTGGTAAGTTTCCGGCC
CCTGGAGATCCCACATAGGTGCCAGGACTCTAGCAGCAAAGATGCACCCACCATTCAGCAGAGCT
CTCCAGGCAGTTCACTTAGACTGGAAGATGAATCTGGAACACCGTCAGAATCATGGCTCTTGTACT
CACACCCAACCAGTAGGAAACAGAGGGTTGACCTAGGTATCTATCTCAACCAGACCCCCTTGGAG
GCTGCCTGCTGGTCCCGCCCCTGGATCTTGCACTGTGGGCCCTGTGGCTACTCTGATCTGGCTGCTC
TGGAGGAGGAGGGCTTGTTTGGGTGTTTGTTTGAATGTGGGACCAAGCAAGAGTGTGAGCAGATT
GCCTTCCGCCTGTTTACACACCGGGAGATCCTGAGTCACCTGCAGGGGGACTGCACCAGCCCTGGT
AGGAACCCAAGCCAATTCAAAAGCAATACGCGTACGCGGCCGCTCGAGCAGAAACTCATCTCAG
AAGAGGATCTGGCAGCAAATGATATCCTGGATTACAAGGATGACGACGATAAGGTTTAA

5 sites were mutated in the 2nd Group 4 motif in NEU3 with the following mutation primers:

Forward CAGTTCACTTAGACTGGAAGATGAATCTGGAACAC

Reverse GTGTTCCAGATTCATCTTCCAGTCTAAGTGAACTG

5. Nucleotide Sequences of Myc-NEU3-Mutant3:

TTTTGTAATACGACTCACTATAGGGCGGCCGGGAATTCGTCGACTGGATCCGGTACCGAGGAGAT
CTGCCGCCGCGATCGCCATGAGACCTGCGGACCTGCCCCCGCGCCCCATGGAAGAATCCCCGGCG
TCCAGCTCTGCCCCGACAGAGACGGAGGAGCCGGGGTCCAGTGCAGAGGTCATGGAAGAAGTGA
CAACATGCTCCTTCAACAGCCCTCTGTTCCGGCAGGAAGATGACAGAGGGATTACCTACCGGATC
CCAGCCCTGCTCTACATACCCCCCACCCACACCTTCCTGGCCTTTGCAGAGAAGCGTTCTACGAGG
AGAGATGAGGATGCTCTCCACCTGGTGCTGAGGCGAGGGTTGAGGATTGGGCAGTTGGTACAGTG
GGGGCCCCTTAAACCACTTATGGAAGCCACACTACCGGGGCATCGGACCATGAACCCCTGTCCTG
TATGGGAGCAGAAGAGTGGTTGTGTGTTCCTGTTCTTCATCTGTGTGCGGGGCCATGTCACAGAGC
GTCAACAGATTGTGTCAGGCAGGAATGCTGCCCGCCTTTGCTTCATCTACAGTCAGGATGCTGGAT
GTTCATGGAGTGAGGTGAGGGACTTGACTGAGGAGGTCATTGGCTCAGAGCTGAAGCACTGGGCC
ACATTTGCTGTGGGCCCAGGTCATGGCATCCAGCTGCAGTCAGGGAGACTGGTCATCCCTGCGTAT
ACCTACTACATCCCTTCCTGGTTCTTTTGCTTCCAGCTACCATGTAAAACCAGGCCTCATTCTCTGA
TGATCTACAGTGATGACCTAGGGGTCACATGGCACCATGGTAGACTCATTAGGCCCATGGTTACA
GTAGAATGTGAAGTGGCAGAGGTGACTGGGAGGGCTGGCCACCCTGTGCTATATTGCAGTGCCCG
GACACCAAACAGGTGCCGGGCAGAGGCGCTCAGCACTGACCATGGTGAAGGCTTTCAGAGACTG
GCCCTGAGTCGACAGCTCTGTGAGCCCCCACATGGTTGCCAAGGGAGTGTGGTAAGTTTCCGGCC
CCTGGAGATCCCACATAGGTGCCAGGACTCTAGCAGCAAAGATGCACCCACCATTCAGCAGAGCT
CTCCAGGCAGTTCACTGAGGCTGGAGGAGGAAGCTGGAACACCGTCAGAATCATGGCTCTTGTAC
TCACACCCAACCAGTAGGAAACAGAGGGTTGACCTAGGTATCTATCTCAACCAGACCCCCTTGGA
GGCTGCCTGCTGGTCCCGCCCCTGGATCTTGCACTGTGGGCCCTGTGGCTACTCTGATCTGGCTGC

TCTGGAGGAGGAGGGCTTGTTTGGGTGTTTGTTTGAATGTGGGACCAAGCAAGAGTGTGAGCAGA
TTGCCTTCCGCCTGTTTACACACCGGGAGATCCTGAGTCACCTGCAGGGGGACTGCACCAGCCCTG
GTAGGAACCCAAGCCAATTCAAAAGCAATACGCGTACGCGGCCGCTC**GAGCAGAAACTCATCTC**
AGAAGAGGATCTGGCAGCAAATGATATCCTG**GATTACAAGGATGACGACGATAAG**GTTTAA

3 sites were mutated in the 1st Group 4 motif in NEU3 with the following mutation primers:

Forward GGGCCCCTTAAACCACTTATGGAAGCCACACTACC

Reverse GGTAGTGTGGCTTCCATAAGTGGTTTAAGGGGCCC

6. qPCR primers for Myc-NEU3 (cover both Myc-Tag and NEU3 sequences)

Forward CAGATTGCCTTCCGCCTGTT

Reverse CTGCCAGATCCTCTTCTGAGAT

b. Myc-ESD plasmids and primers

1. Plasmid map of Myc-ESD-WT

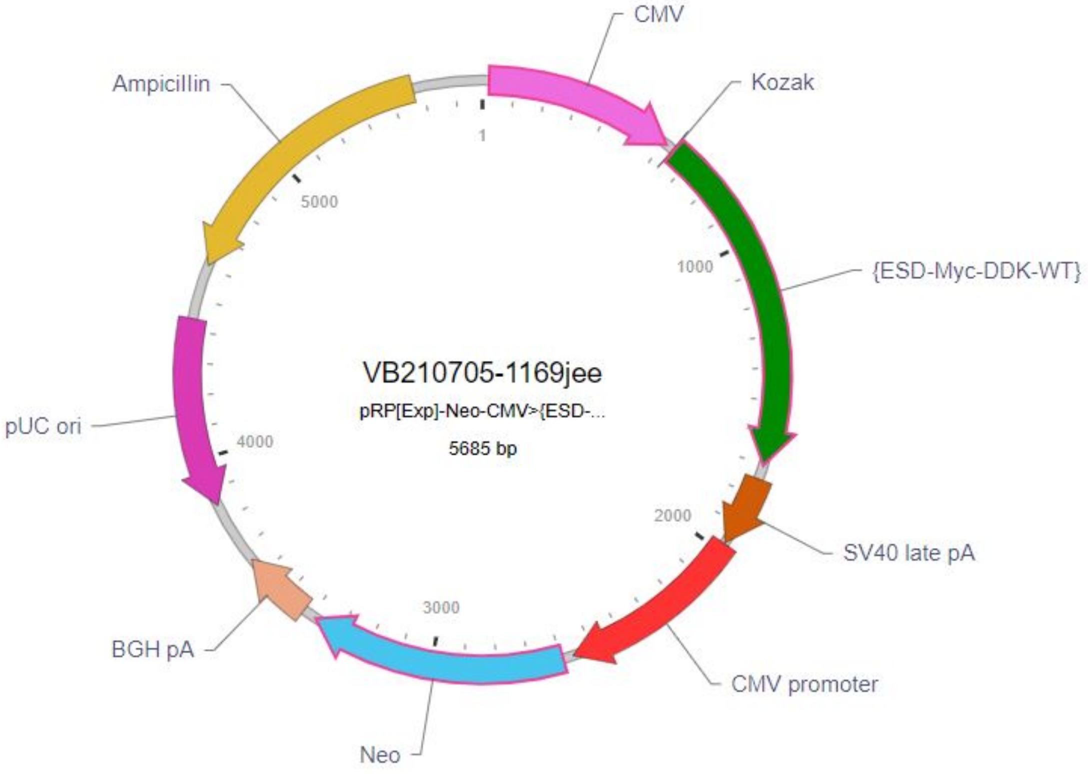

2. Nucleotide Sequences of Myc-ESD-WT:

aaagcgagagtgagtgggaccggaggggcggggcatcatatgggcggggctgaggcgaggccccggcggccatcttgagccccgccttttac
ttcggcccgcttcttctggtcactccgccaccgtagaatcgcctaccatttggtgcaagcaaaaagcaatcagcaattggacaggaaaagaATG
GAGCAGAAACTCATCTCAGAAGAGGATCTGGATTACAAGGATGACGACGATAAGGCATT
GAAGCAGATTTCCAGCAACAAGTGCTTTGGGGGATTGCAGAAAGTTTTTGAACATGACA
GTGTTGAACTAAACTGCAAAATGAAATTTGCTGTCTACTTACCACCAAAGGCAGAAACA
GGAAAGTGCCCTGCACTGTATTGGCTCTCAGGTTTAACTTGCACAGAGCAAAATTTTATA
TCAAAATCTGGTTATCATCAGTCTGCTTCAGAACATGGTCTTGTTGTCATTGCTCCAGATA
CCAGCCCTCGTGGCTGCAATATTAAAGGTGAAGATGAGAGCTGGGACTTTGGCACTGGT
GCTGGATTTTATGTTGATGCCACTGAAGATCCTTGGAAAACCAACTACAGAATGTACTCT
TATGTCACAGAGGAGCTTCCCCAACTCATAAATGCCAATTTTCCAGTGGATCCCCAAAGG
ATGTCTATTTTTGGCCACTCCATGGGAGGTCATGGAGCTCTGATCTGTGCTTTGAAAAAT
CCTGGAAAATACAAATCTGTGTCAGCATTTGCTCCAATTTGCAACCCTGTACTCTGTCCC
TGGGGCAAAAAAGCCTTTAGTGGATATTTGGGAACAGATCAAAGTAAATGGAAGGCTTA
TGATGCTACCCACCTTGTGAAATCCTATCCAGGATCTCAGCTGGACATACTAATTGATCA
AGGGAAAGATGACCAGTTTCTTTTAGATGGACAGTTACTCCCTGATAACTTCATAGCTGC
CTGTACAGAAAAGAAAATCCCCGTTGTTTTTCGATTGCAAGAGGGTTATGATCATAGCTA
CTACTTCATTGCAACCTTTATTACTGACCACATCAGACATCATGCTAAATACCTGAATGC

ATGAaaaaactccaaataagagaatctcttcaggattataaaagttgtaaaatgcaactgtattgctgagcaaaaaaaaaaaaaaattcaaaacatt
ggattttatagtgctaaaagggctttattctatagttgaatcacctctgaataaagatataaaaccta

A Myc-Tag is inserted in frame with the coding sequence of ESD.

3. Nucleotide Sequences of Myc-ESD-Motif in CDS:

aaagcgagagtgagtgggaccggaggggcggggcatcatatgggcggggctgaggcgaggccccggcggccatcttgagccccgccttttac
ttcggcccgcttcttctggtcactccgccaccgtagaatcgcctaccatttggtgcaagcaaaaagcaatcagcaattggacaggaaaagaATG
GAGCAGAAACTCATCTCAGAAGAGGATCTGGATTACAAGGATGACGACGATAAGGCATT
GAAGCAGATTTCCAGCAACAAGTGCTTTGGGGGATTGCAGAAAGTTTTTGAACATGACA
GTGTTGAACTAAACTGCAAAATGAAATTTGCTGTCTACTTACCACCAAAGGCAGAAACA
GGAAAGTGCCCTGCACTGTATTGGCTCTCAGGTTTAACTTGCACAGAGCAAAATTTTATA
TCAAAATCTGGTTATCATCAGTCTGCTTCAGAACATGGTCTTGTTGTCATTGCTCCAGATA
CCAGCCCTCGTGGCTGCAATATTAAAGGTGAAGATGAGAGCTGGGACTTTGGCACTGGT
GCTGGATTTTATGTTGATGCCACTGAAGATCCTTGGAAAACCAACTACAGAATGTACTCT
TATGTCACAGAGGAGCTTCCCCAACTCATAAATGCCAATTTTCCAGTGGATCCCCAAAGG
ATGTCTATTTTTGGAGGAGGAGGAGGAGGAGGCCACTCCATGGGAGGTCATGGAGCTCT
GATCTGTGCTTTGAAAAATCCTGGAAAATACAAATCTGTGTCAGCATTTGCTCCAATTTG
CAACCCTGTACTCTGTCCCTGGGGCAAAAAAGCCTTTAGTGGATATTTGGGAACAGATCA
AAGTAAATGGAAGGCTTATGATGCTACCCACCTTGTGAAATCCTATCCAGGATCTCAGCT
GGACATACTAATTGATCAAGGGAAAGATGACCAGTTTCTTTTAGATGGACAGTTACTCCC
TGATAACTTCATAGCTGCCTGTACAGAAAAGAAAATCCCCGTTGTTTTTCGATTGCAAGA
GGGTTATGATCATAGCTACTACTTCATTGCAACCTTTATTACTGACCACATCAGACATCA
TGCTAAATACCTGAATGCATGAaaaaactccaaataagagaatctcttcaggattataaaagttgtaaaatgcaactgtattgct
gagcaaaaaaaaaaaaaaattcaaaacattggattttatagtgctaaaagggctttattctatagttgaatcacctctgaataaagatataaaaccta

A Myc-Tag and a Group 4 motif are inserted in frame with the coding sequence of ESD.

4. Nucleotide Sequences of Myc-ESD-Motif in 3’UTR:

aaagcgagagtgagtgggaccggaggggcggggcatcatatgggcggggctgaggcgaggccccggcggccatcttgagccccgccttttac
ttcggcccgcttcttctggtcactccgccaccgtagaatcgcctaccatttggtgcaagcaaaaagcaatcagcaattggacaggaaaagaATG
GAGCAGAAACTCATCTCAGAAGAGGATCTGGATTACAAGGATGACGACGATAAGGCATT
GAAGCAGATTTCCAGCAACAAGTGCTTTGGGGGATTGCAGAAAGTTTTTGAACATGACA
GTGTTGAACTAAACTGCAAAATGAAATTTGCTGTCTACTTACCACCAAAGGCAGAAACA
GGAAAGTGCCCTGCACTGTATTGGCTCTCAGGTTTAACTTGCACAGAGCAAAATTTTATA
TCAAAATCTGGTTATCATCAGTCTGCTTCAGAACATGGTCTTGTTGTCATTGCTCCAGATA
CCAGCCCTCGTGGCTGCAATATTAAAGGTGAAGATGAGAGCTGGGACTTTGGCACTGGT
GCTGGATTTTATGTTGATGCCACTGAAGATCCTTGGAAAACCAACTACAGAATGTACTCT
TATGTCACAGAGGAGCTTCCCCAACTCATAAATGCCAATTTTCCAGTGGATCCCCAAAGG
ATGTCTATTTTTGGCCACTCCATGGGAGGTCATGGAGCTCTGATCTGTGCTTTGAAAAAT
CCTGGAAAATACAAATCTGTGTCAGCATTTGCTCCAATTTGCAACCCTGTACTCTGTCCC
TGGGGCAAAAAAGCCTTTAGTGGATATTTGGGAACAGATCAAAGTAAATGGAAGGCTTA
TGATGCTACCCACCTTGTGAAATCCTATCCAGGATCTCAGCTGGACATACTAATTGATCA
AGGGAAAGATGACCAGTTTCTTTTAGATGGACAGTTACTCCCTGATAACTTCATAGCTGC

CTGTACAGAAAAGAAAATCCCCGTTGTTTTTCGATTGCAAGAGGGTTATGATCATAGCTA
CTACTTCATTGCAACCTTTATTACTGACCACATCAGACATCATGCTAAATACCTGAATGC
ATGAaaaaactccaaataagagaatctcttcaggattataaaagttgtaaaatgcaactgtattgctgagcaaaaaaaaaaaaaattcaaaacatt
GGAGGAGGAGGAGGAGGAggattttatagtgctaaaagggctttattctatagttgaatcacctctgaataaagatataaaaccta

A Myc-Tag is inserted in frame with the coding sequence of ESD. A Group 4 motif is inserted into the 3’UTR of ESD.

5. qPCR primers for Myc-ESD (cover both Myc-Tag and ESD sequences)

Forward GGATCTGGATTACAAGGATGAC

Reverse CTGCCTTTGGTGGTAAGTAGAC

c. Myc-MKKS plasmids and primers

1. Plasmid map of Myc-MKKS-WT

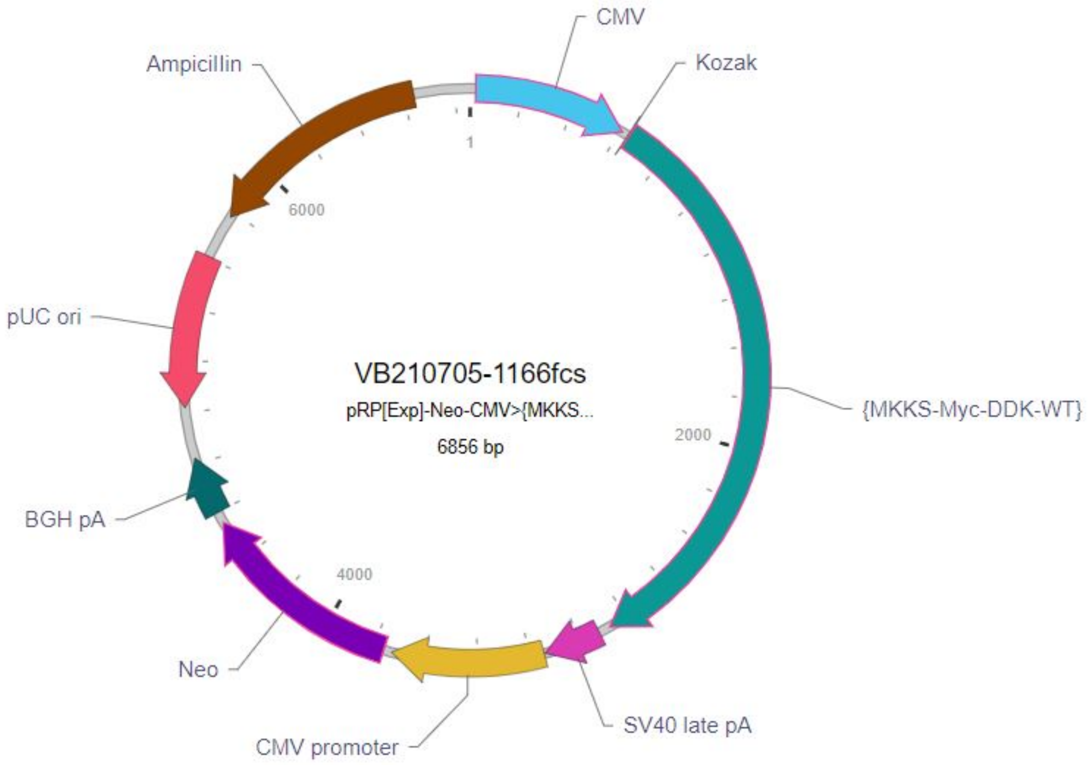

2. Nucleotide Sequences of Myc-MKKS-WT:

agagctgcgcgtgctccgtgccctcgcgcgacgcgaaggttgtcgggatccgcggcagcagcggctgcttgagatctgtttctggggcctctggc
ggtggcggcctggggcggcgcgacggctggtgcgcaggtacactgatgctgaagtactatgagccttcggaacttgtggagagactacaaagttt
tggttgttatggtccctttagttgggctcatacatttggggtggtacagaatcaaaagcagccctgttttccaaatacctaaaaacgacgacattcctga
gcaagatagtctgggactttcaaatcttcagaagagccaaatccaggggaagtagcaggcttgcaatcttcaggtaaagaagcagctttgaatctga
gcttcatatcgaaagaagagatgaaaaataccagttggattagaaagaactggcttcttgtagctgggatatctttcataggtgtccatcttggaacata
ctttttgcagaggtctgcaaagcagtctgtaaaatttcagtctcaaagcaaacaaaagagtattgaagagtgaagtaaaataaatatttggaattactaa
tttgtcattaaatcattctatgctgattagcttcataaacattgaactttttgattttatagccacaatgctgcatattcatactttaattcctaaagaataattttt
aatgttaaaacgtgataatgcaataaatagaaaaatgtggtttacaaaataaaaacggtcttcactagttaccacctgaagtaagATGGAGCA
GAAACTCATCTCAGAAGAGGATCTGGATTACAAGGATGACGACGATAAGTCTCGTTTGG
AAGCTAAGAAGCCATCATTGTGTAAGAGTGAACCACTGACAACTGAGAGAGTCAGGACC
ACACTTTCTGTCTTGAAAAGAATTGTAACATCATGCTATGGCCCCTCAGGTAGGCTGAAG
CAGCTGCACAATGGCTTTGGAGGTTACGTGTGTACAACCTCACAGTCCTCAGCTCTGCTC
AGTCACCTTTTGGTCACACATCCCATTTTAAAGATCCTGACAGCCTCCATACAGAATCAT
GTGTCAAGCTTCAGTGATTGTGGCTTATTCACAGCTATTCTTTGCTGCAACCTGATTGAA
AATGTTCAGAGATTAGGCTTGACACCCACCACTGTCATTAGATTAAATAAACATCTTTTG
AGTCTTTGCATCAGTTATCTCAAGTCTGAGACCTGTGGTTGTCGAATCCCAGTGGACTTT
AGTAGTACTCAGATCCTCCTTTGTTTGGTGCGTAGTATATTAACAAGTAAACCTGCCTGT

ATGCTCACCAGAAAGGAAACAGAGCATGTCAGTGCTTTGATCCTGAGAGCCTTTTTGCTT
ACAATTCCAGAAAATGCTGAAGGCCACATCATTTTAGGAAAGAGTTTAATTGTACCTTTA
AAAGGTCAAAGAGTTATAGATTCCACTGTATTACCTGGGATACTCATTGAAATGTCAGA
AGTTCAATTAATGAGGCTATTACCTATCAAAAAATCAACTGCCCTCAAGGTGGCACTCTT
TTGTACAACTTTATCCGGAGACACTTCTGACACTGGAGAAGGAACTGTGGTGGTCAGTTA
TGGGGTTTCTCTTGAAAATGCAGTCTTGGACCAGCTGCTTAACCTAGGAAGGCAGCTAAT
CAGTGACCACGTAGATCTTGTCCTGTGCCAAAAAGTTATACATCCATCTTTGAAGCAGTT
TCTCAATATGCATCGTATTATTGCCATAGACAGAATTGGAGTGACTCTGATGGAACCCCT
GACTAAAATGACAGGAACACAGCCTATTGGATCCCTAGGCTCAATATGTCCTAATAGTT
ATGGAAGTGTGAAAGATGTGTGCACTGCAAAATTTGGCTCCAAACATTTTTTTCATCTTA
TTCCTAATGAAGCAACAATCTGCAGCTTGCTTCTCTGCAACAGAAATGACACTGCCTGGG
ATGAGCTGAAGCTCACGTGTCAGACGGCACTGCATGTCCTGCAGTTAACACTCAAGGAA
CCATGGGCTTTGTTGGGAGGTGGCTGTACTGAAACTCATTTGGCTGCATATATCAGACAC
AAGACTCACAACGACCCAGAAAGCATTCTCAAAGATGATGAATGTACTCAAACAGAACT
TCAATTAATTGCTGAAGCATTTTGCAGTGCCCTAGAATCTGTTGTTGGCTCTTTAGAACAT
GATGGAGGTGAAATTCTCACTGACATGAAGTATGGACACCTTTGGTCAGTTCAGGCAGA
TTCTCCCTGTGTTGCTAACTGGCCAGATTTGCTTTCACAGTGTGGCTGTGGATTATACAAT
AGCCAGGAAGAACTCAACTGGTCTTTCTTAAGAAGCACACGTCGTCCATTTGTGCCACAA
AGCTGCCTTCCACATGAAGCTGTGGGCTCAGCCAGCAACCTGACCTTGGACTGTTTGACT
GCAAAGCTTAGTGGCCTACAGGTGGCTGTAGAGACAGCCAATTTGATTTTGGATCTTTCA
TATGTTATTGAAGATAAAAACTAAgagaatagcatgttcgtattacaagagaaacaaataaactagtctgttggcaattgaga
aaaattgtgagtgtatttgttttctcccaaagccctgttctacatatttggacaaatgactcataaaattatagatacacttatttaggaaaaaaggtgattc
gtgaatggaaatgccatgaaacaataaaaatatgaagcattatttatttaaaaatattatagttatcttagggattctatactggctgctgtacattgttcta
aatttttgttatgttggcatcattttgagagcaaacaaataaaaaagactcctaatccatgctctagtttggatatacatattttagatattttccagttagca
gtaattacatatgcttaaagtataaaactagatctcaaagtgtcacaaaacattcagtatatattgctcaatcaaaagaagtattcaaactgcacatt

A Myc-Tag is inserted in frame with the coding sequence of MKKS.

3. Nucleotide Sequences of Myc-MKKS-Motif in CDS:

agagctgcgcgtgctccgtgccctcgcgcgacgcgaaggttgtcgggatccgcggcagcagcggctgcttgagatctgtttctggggcctctggc
ggtggcggcctggggcggcgcgacggctggtgcgcaggtacactgatgctgaagtactatgagccttcggaacttgtggagagactacaaagttt
tggttgttatggtccctttagttgggctcatacatttggggtggtacagaatcaaaagcagccctgttttccaaatacctaaaaacgacgacattcctga
gcaagatagtctgggactttcaaatcttcagaagagccaaatccaggggaagtagcaggcttgcaatcttcaggtaaagaagcagctttgaatctga
gcttcatatcgaaagaagagatgaaaaataccagttggattagaaagaactggcttcttgtagctgggatatctttcataggtgtccatcttggaacata
ctttttgcagaggtctgcaaagcagtctgtaaaatttcagtctcaaagcaaacaaaagagtattgaagagtgaagtaaaataaatatttggaattactaa
tttgtcattaaatcattctatgctgattagcttcataaacattgaactttttgattttatagccacaatgctgcatattcatactttaattcctaaagaataattttt
aatgttaaaacgtgataatgcaataaatagaaaaatgtggtttacaaaataaaaacggtcttcactagttaccacctgaagtaagATGGAGCA
GAAACTCATCTCAGAAGAGGATCTGGATTACAAGGATGACGACGATAAGTCTCGTTTGG
AAGCTAAGAAGCCATCATTGTGTAAGAGTGAACCACTGACAACTGAGAGAGTCAGGACC
ACACTTTCTGTCTTGAAAAGAATTGTAACATCATGCTATGGCCCCTCAGGTAGGCTGAAG
CAGCTGCACAATGGCTTTGGAGGTTACGTGTGTACAACCTCACAGTCCTCAGCTCTGCTC
AGTCACCTTTTGGTCACACATCCCATTTTAAAGATCCTGACAGCCTCCATACAGAATCAT
GTGTCAAGCTTCAGTGATTGTGGAGGAGGAGGAGGAGGAGGCTTATTCACAGCTATTCT
TTGCTGCAACCTGATTGAAAATGTTCAGAGATTAGGCTTGACACCCACCACTGTCATTAG
ATTAAATAAACATCTTTTGAGTCTTTGCATCAGTTATCTCAAGTCTGAGACCTGTGGTTGT
CGAATCCCAGTGGACTTTAGTAGTACTCAGATCCTCCTTTGTTTGGTGCGTAGTATATTA

ACAAGTAAACCTGCCTGTATGCTCACCAGAAAGGAAACAGAGCATGTCAGTGCTTTGAT
CCTGAGAGCCTTTTTGCTTACAATTCCAGAAAATGCTGAAGGCCACATCATTTTAGGAAA
GAGTTTAATTGTACCTTTAAAAGGTCAAAGAGTTATAGATTCCACTGTATTACCTGGGAT
ACTCATTGAAATGTCAGAAGTTCAATTAATGAGGCTATTACCTATCAAAAAATCAACTGC
CCTCAAGGTGGCACTCTTTTGTACAACTTTATCCGGAGACACTTCTGACACTGGAGAAGG
AACTGTGGTGGTCAGTTATGGGGTTTCTCTTGAAAATGCAGTCTTGGACCAGCTGCTTAA
CCTAGGAAGGCAGCTAATCAGTGACCACGTAGATCTTGTCCTGTGCCAAAAAGTTATAC
ATCCATCTTTGAAGCAGTTTCTCAATATGCATCGTATTATTGCCATAGACAGAATTGGAG
TGACTCTGATGGAACCCCTGACTAAAATGACAGGAACACAGCCTATTGGATCCCTAGGC
TCAATATGTCCTAATAGTTATGGAAGTGTGAAAGATGTGTGCACTGCAAAATTTGGCTCC
AAACATTTTTTTCATCTTATTCCTAATGAAGCAACAATCTGCAGCTTGCTTCTCTGCAACA
GAAATGACACTGCCTGGGATGAGCTGAAGCTCACGTGTCAGACGGCACTGCATGTCCTG
CAGTTAACACTCAAGGAACCATGGGCTTTGTTGGGAGGTGGCTGTACTGAAACTCATTTG
GCTGCATATATCAGACACAAGACTCACAACGACCCAGAAAGCATTCTCAAAGATGATGA
ATGTACTCAAACAGAACTTCAATTAATTGCTGAAGCATTTTGCAGTGCCCTAGAATCTGT
TGTTGGCTCTTTAGAACATGATGGAGGTGAAATTCTCACTGACATGAAGTATGGACACCT
TTGGTCAGTTCAGGCAGATTCTCCCTGTGTTGCTAACTGGCCAGATTTGCTTTCACAGTGT
GGCTGTGGATTATACAATAGCCAGGAAGAACTCAACTGGTCTTTCTTAAGAAGCACACG
TCGTCCATTTGTGCCACAAAGCTGCCTTCCACATGAAGCTGTGGGCTCAGCCAGCAACCT
GACCTTGGACTGTTTGACTGCAAAGCTTAGTGGCCTACAGGTGGCTGTAGAGACAGCCA
ATTTGATTTTGGATCTTTCATATGTTATTGAAGATAAAAACTAAgagaatagcatgttcgtattacaagag
aaacaaataaactagtctgttggcaattgagaaaaattgtgagtgtatttgttttctcccaaagccctgttctacatatttggacaaatgactcataaaatt
atagatacacttatttaggaaaaaaggtgattcgtgaatggaaatgccatgaaacaataaaaatatgaagcattatttatttaaaaatattatagttatctt
agggattctatactggctgctgtacattgttctaaatttttgttatgttggcatcattttgagagcaaacaaataaaaaagactcctaatccatgctctagttt
ggatatacatattttagatattttccagttagcagtaattacatatgcttaaagtataaaactagatctcaaagtgtcacaaaacattcagtatatattgctc
aatcaaaagaagtattcaaactgcacatt

A Myc-Tag and a Group 4 motif are inserted in frame with the coding sequence of MKKS.

4. Nucleotide Sequences of Myc-ESD-Motif in 3'UTR:

agagctgcgcgtgctccgtgccctcgcgcgacgcgaaggttgtcgggatccgcggcagcagcggctgcttgagatctgtttctggggcctctggc
ggtggcggcctggggcggcgcgacggctggtgcgcaggtacactgatgctgaagtactatgagccttcggaacttgtggagagactacaaagttt
tggttgttatggtccctttagttgggctcatacatttggggtggtacagaatcaaaagcagccctgttttccaaatacctaaaaacgacgacattcctga
gcaagatagtctgggactttcaaatcttcagaagagccaaatccaggggaagtagcaggcttgcaatcttcaggtaaagaagcagctttgaatctga
gcttcatatcgaaagaagagatgaaaaataccagttggattagaaagaactggcttcttgtagctgggatatctttcataggtgtccatcttggaacata
ctttttgcagaggtctgcaaagcagtctgtaaaatttcagtctcaaagcaaacaaaagagtattgaagagtgaagtaaaataaatatttggaattactaa
tttgtcattaaatcattctatgctgattagcttcataaacattgaactttttgattttatagccacaatgctgcatattcatactttaattcctaaagaataattttt
aatgttaaaacgtgataatgcaataaatagaaaaatgtggtttacaaaataaaaacggtcttcactagttaccacctgaagtaagATGGAGCA
GAAACTCATCTCAGAAGAGGATCTGGATTACAAGGATGACGACGATAAGTCTCGTTTGG
AAGCTAAGAAGCCATCATTGTGTAAGAGTGAACCACTGACAACTGAGAGAGTCAGGACC
ACACTTTCTGTCTTGAAAAGAATTGTAACATCATGCTATGGCCCCTCAGGTAGGCTGAAG
CAGCTGCACAATGGCTTTGGAGGTTACGTGTGTACAACCTCACAGTCCTCAGCTCTGCTC
AGTCACCTTTTGGTCACACATCCCATTTTAAAGATCCTGACAGCCTCCATACAGAATCAT
GTGTCAAGCTTCAGTGATTGTGGCTTATTCACAGCTATTCTTTGCTGCAACCTGATTGAA
AATGTTCAGAGATTAGGCTTGACACCCACCACTGTCATTAGATTAAATAAACATCTTTTG

AGTCTTTGCATCAGTTATCTCAAGTCTGAGACCTGTGGTTGTCGAATCCCAGTGGACTTT
AGTAGTACTCAGATCCTCCTTTGTTTGGTGCGTAGTATATTAACAAGTAAACCTGCCTGT
ATGCTCACCAGAAAGGAAACAGAGCATGTCAGTGCTTTGATCCTGAGAGCCTTTTTGCTT
ACAATTCCAGAAAATGCTGAAGGCCACATCATTTTAGGAAAGAGTTTAATTGTACCTTTA
AAAGGTCAAAGAGTTATAGATTCCACTGTATTACCTGGGATACTCATTGAAATGTCAGA
AGTTCAATTAATGAGGCTATTACCTATCAAAAAATCAACTGCCCTCAAGGTGGCACTCTT
TTGTACAACTTTATCCGGAGACACTTCTGACACTGGAGAAGGAACTGTGGTGGTCAGTTA
TGGGGTTTCTCTTGAAAATGCAGTCTTGGACCAGCTGCTTAACCTAGGAAGGCAGCTAAT
CAGTGACCACGTAGATCTTGTCCTGTGCCAAAAAGTTATACATCCATCTTTGAAGCAGTT
TCTCAATATGCATCGTATTATTGCCATAGACAGAATTGGAGTGACTCTGATGGAACCCCT
GACTAAAATGACAGGAACACAGCCTATTGGATCCCTAGGCTCAATATGTCCTAATAGTT
ATGGAAGTGTGAAAGATGTGTGCACTGCAAAATTTGGCTCCAAACATTTTTTTCATCTTA
TTCCTAATGAAGCAACAATCTGCAGCTTGCTTCTCTGCAACAGAAATGACACTGCCTGGG
ATGAGCTGAAGCTCACGTGTCAGACGGCACTGCATGTCCTGCAGTTAACACTCAAGGAA
CCATGGGCTTTGTTGGGAGGTGGCTGTACTGAAACTCATTTGGCTGCATATATCAGACAC
AAGACTCACAACGACCCAGAAAGCATTCTCAAAGATGATGAATGTACTCAAACAGAACT
TCAATTAATTGCTGAAGCATTTTGCAGTGCCCTAGAATCTGTTGTTGGCTCTTTAGAACAT
GATGGAGGTGAAATTCTCACTGACATGAAGTATGGACACCTTTGGTCAGTTCAGGCAGA
TTCTCCCTGTGTTGCTAACTGGCCAGATTTGCTTTCACAGTGTGGCTGTGGATTATACAAT
AGCCAGGAAGAACTCAACTGGTCTTTCTTAAGAAGCACACGTCGTCCATTTGTGCCACAA
AGCTGCCTTCCACATGAAGCTGTGGGCTCAGCCAGCAACCTGACCTTGGACTGTTTGACT
GCAAAGCTTAGTGGCCTACAGGTGGCTGTAGAGACAGCCAATTTGATTTTGGATCTTTCA
TATGTTATTGAAGATAAAAACTAAgagaatagcatgttcgtattacaagagaaacaaataaactagtctgttggcaattgaga
aaaattgtgagtgtatttgttttctcccaaagccctgttctacatatttGGAGGAGGAGGAGGAGGAggacaaatgactcataaaatta
tagatacacttatttaggaaaaaaggtgattcgtgaatggaaatgccatgaaacaataaaaatatgaagcattatttatttaaaaatattatagttatctta
gggattctatactggctgctgtacattgttctaaatttttgttatgttggcatcattttgagagcaaacaaataaaaaagactcctaatccatgctctagttt
ggatatacatattttagatattttccagttagcagtaattacatatgcttaaagtataaaactagatctcaaagtgtcacaaaacattcagtatatattgctc
aatcaaaagaagtattcaaactgcacatt

A Myc-Tag is inserted in frame with the coding sequence of MKKS. A Group 4 motif is inserted into the 3’UTR of MKKS.

5. qPCR primers for Myc-MKKS (cover both Myc-Tag and MKKS sequences)

Forward CATCTCAGAAGAGGATCTGG

Reverse CAAGACAGAAAGTGTGGTCC

d. Biotin labeled Group 4 Motif

1. Biotin-Group 4 Motif sequence:

/5Biosg/ GGAGGAGGAGGAGGAGGAGG

2. 5' Biotin information:

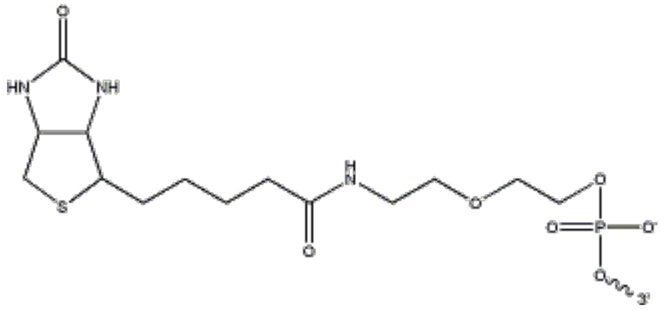

MW: 393.4
